# Gc inhibition preserves insulin sensitivity and reduces body weight without loss of muscle mass

**DOI:** 10.1172/jci.insight.195341

**Published:** 2025-12-08

**Authors:** Richard Gill, Taiyi Kuo

**Affiliations:** Department of Neurobiology, Physiology, and Behavior, University of California Davis, Davis, USA.

**Keywords:** Endocrinology, Metabolism, Diabetes, Obesity

## Abstract

Obesity and type 2 diabetes (T2D) are metabolic diseases with increasing prevalence worldwide. Obesity often leads to T2D. Insulin resistance and impaired β cell function contribute to the onset of hyperglycemia. Previously, we reported that ablation of *Gc*, encoding a secreted protein with a primary role in vitamin D transport, improved pancreatic β cell function in models of diet-induced insulin resistance. Here, we show that *Gc* ablation had systemic insulin-sensitizing effects to prevent weight gain, hyperglycemia, and glucose intolerance; lower nonesterified fatty acids and triglycerides; and augment glucose uptake in skeletal muscle and adipose in male mice fed a high-fat diet. Interestingly, weight loss in *Gc*-ablated mice resulted from selective fat mass loss with preserved lean mass. Moreover, acute *Gc* inhibition prevented glucose intolerance caused by high-fat feeding. The data suggest that *Gc* inhibition can increase insulin production in β cells and insulin action in peripheral tissues, while reducing fat mass.

## Abstract 2

Gc inhibition improves insulin secretion and sensitivity, without losing muscle or suppressing appetite, suggesting that Gc inhibition may be an ideal target for diabetes therapies.

## Graphical abstract

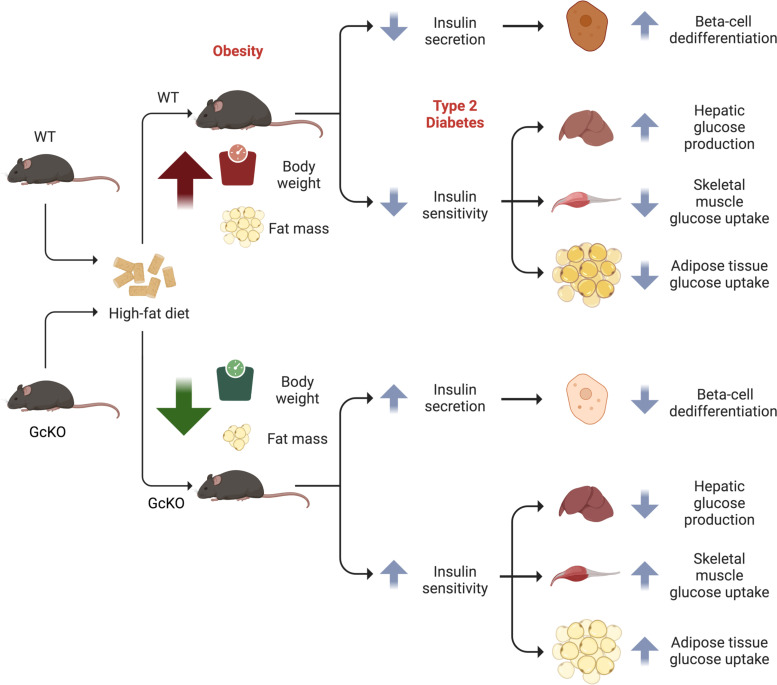

## Introduction

Obesity, characterized by accumulation of body fat, is a major risk factor for type 2 diabetes (T2D). Fueled by sedentary lifestyle and unhealthy dietary patterns, the prevalence of both obesity and T2D have increased rapidly worldwide, resulting in a double pandemic. Insulin resistance and insulin deficiency due to β cell failure underlie most forms of T2D, and efforts to treat diabetes have therefore been directed toward increasing insulin sensitivity and insulin production (1). During the natural history of T2D, insulin resistance possibly predates β cell abnormalities but remains relatively constant (2, 3), whereas β cell function deteriorates rapidly following the onset of hyperglycemia, as a result of β cells’ failure to increase in mass and produce more insulin in response to insulin resistance (4, 5). Advances in the understanding of the pathophysiology of the disease have shaped diabetes treatment: combinations of agents that improve β cell function with those that decrease insulin resistance are now considered standard therapy (6). In recent years, GLP1R agonist–based approaches coupled with diet and exercise have helped patients to lose up to ~20% of their body weight by increasing insulin secretion in the pancreatic β cells, increasing insulin sensitivity, and reducing appetite. However, there are significant adverse gastrointestinal manifestations with GLP-1 agonists. Also, treatment-associated weight loss is a result of muscle, bone, and adipose tissue reduction. An ideal therapy would reduce fat but not in muscle and bone mass, while minimizing the above treatment-associated side effects that are likely associated with appetite suppression.

Here, we demonstrate that Gc inhibition improves metabolic health without affecting appetite or muscle mass in mice. *Gc* ablation was previously shown to have no effect on bone mass (7). Moreover, we show that Gc can be effectively targeted and knocked down using an adenoviral system in mice, as it is expressed primarily in the liver and is secreted into the bloodstream. Since its discovery in 1959 (8), Gc has been shown to serve 2 major functions: (a) to transport all vitamin D metabolites and (b) to bind to globular actin to form a Gc-actin complex that prevents actin repolymerization in serum after tissue damage (9). Interestingly, its expression in the pancreas is limited to glucagon-producing α cells (10). However, we discovered that, during the progression of multiparity-, age-, or diet-induced diabetes in mice, *Gc* expression is ectopically activated in β cells (11). Moreover, in the absence of β cell transcription factors Pax6 or Isl1, *Gc* expression increases in β cells or whole islets, respectively (12, 13). While its function in the endocrine islet remains unclear, genetic KO of *Gc* preserved insulin secretory response and β cell function in vivo, as determined by hyperglycemic clamps, and ex vivo in isolated islets (11). Based on human cross-sectional studies, *GC* variants have been associated with increased fasting glucose and insulin levels, as well as impaired oral glucose tolerance tests (14–20). *GC* polymorphisms have also been associated with increased susceptibility to T2D (18), obesity (20–25), and dyslipidemia (20, 26, 27). However, these associations provide limited information regarding disease-relevant cell type(s) and mechanism of action. Therefore, the pathophysiological relationships between GC, diabetes, and obesity remain largely unknown. Two individuals nullizygous for *GC* were identified in case reports from 2019 and 2021 (28, 29), providing evidence that absence of GC is not lethal in humans. However, the metabolic statuses of these individuals have not been investigated.

In the course of experiments using high-fat diet (HFD) to induce insulin resistance and hyperglycemia and to investigate Gc function in the endocrine pancreatic islet, we observed that *Gc*-deficient mice gained less weight than their control WT littermates. This observation prompted us to conduct an in-depth investigation into the metabolic effects of genetic *Gc* ablation on systemic insulin sensitivity and insulin action. Here, we show that, in addition to improving β cell function, *Gc* deletion preserves insulin sensitivity in diet-induced obesity. In hyperinsulinemic-euglycemic clamps, whole-body Gc knockout (GcKO) increases the suppression of hepatic glucose production by insulin, glucose turnover, and glucose uptake in skeletal muscle and adipose tissue. Moreover, indirect calorimetry studies show increased energy expenditure in GcKO mice. Furthermore, HFD-fed GcKO mice show lower fasting glycemia and improved glycemia during glucose and pyruvate tolerance tests, even when their body weight, fat mass, and lean mass are matched to WT. Therefore, Gc inhibition in mice induces weight loss without suppressing appetite and preserves muscle mass while reducing fat mass, suggesting Gc inhibition as a promising potential therapeutic avenue for the treatment of obesity and T2D.

## Results

### Metabolic profiles of GcKO mice.

**Figure 1:**
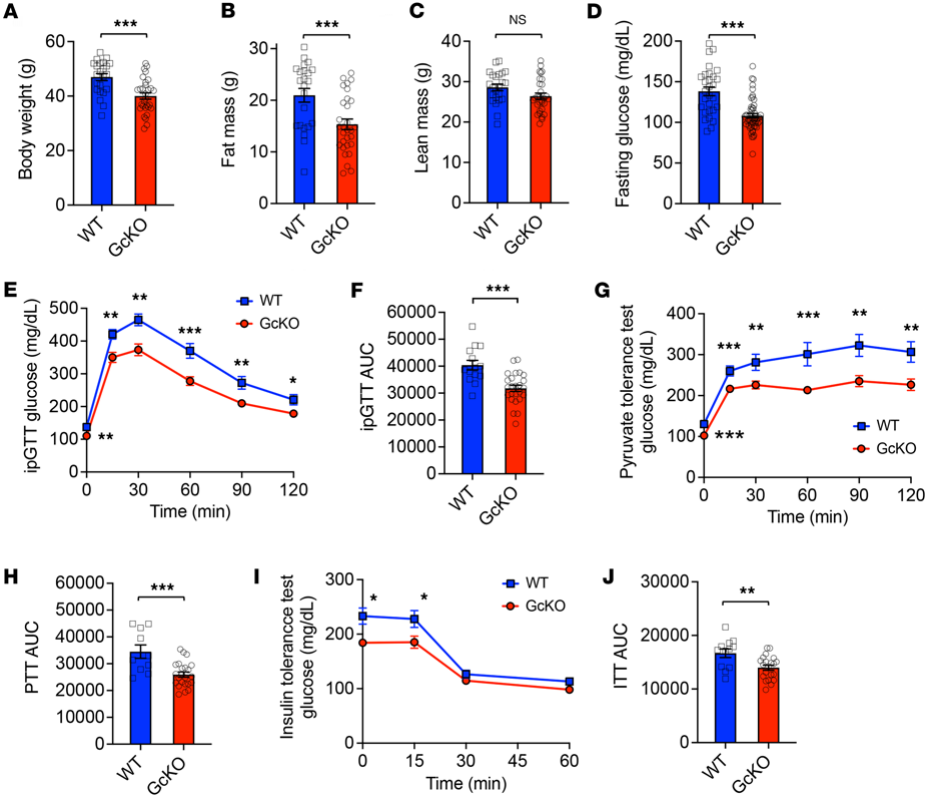
Metabolic features of HFD-fed GcKO male mice. (**A–C**) Body weight (WT *n* = 23, GcKO *n* = 30), fat mass (WT *n* = 23, GcKO *n* = 30), and lean mass (WT *n* = 23, GcKO *n* = 30) of HFD-fed WT and GcKO mice. (**D**) Fasting glycemia (WT *n* = 29, GcKO *n* = 53) of HFD-fed WT and GcKO mice. (**E** and **F**) I.p. glucose tolerance tests (ipGTT) performed and AUC calculated in HFD-fed WT (*n* = 14) and GcKO (*n* 25) mice. (**G**) Pyruvate tolerance test (PTT) performed and (**H**) AUC calculated in HFD-fed WT (*n* = 10) and GcKO (*n* = 23) mice. (**I**) Insulin tolerance test (ITT) performed and (**J**) AUC calculated in HFD-fed WT (WT = 8) and GcKO (*n* = 16) mice. “NS” indicates no significant statistical difference. Data are shown as mean ± SEM, *P < 0.05, **P < 0.01, ***P < 0.005 by Student’s *t* test.

We placed WT and GcKO mice on a HFD as a metabolic challenge. Following 12 weeks of HFD, GcKO mice showed ~15% lower body weight compared with WT (46.9 ± 1.3 versus 40.0 ± 1.2 g; *P* < 0.001) (Figure 1A). We utilized dual-energy X-ray absorptiometry to determine body composition. We found that the lower weight of GcKO mice is attributable to a 27% decrease in fat mass (21.0 ± 1.3 versus 15.3 ± 1.0 g; *P* < 0.005) (Figure 1B), as lean mass did not differ from that of WT mice (28.6 ± 0.8 versus 26.4 ± 0.8 g) (Figure 1C).

To evaluate the consequences of *Gc* ablation on glucose metabolism, we performed glucose tolerance tests in WT and GcKO mice. GcKO mice showed a ~22% decrease in fasting glycemia compared with WT (138.1 ± 5.4 versus 108.1 ± 2.7 mg/dL; *P* < 0.001) (Figure 1D). We observed a significant improvement in glucose tolerance in HFD-fed male GcKO mice compared with WT throughout the 2-hour test (Figure 1, E and F). GcKO mice on chow diet displayed lowered fasting glucose (92 ± 4.1 versus 74 ± 2.7 mg/dL; *P* < 0.001) but not glucose tolerance compared with WT (Supplemental Figure 1, A–D; supplemental material available online with this article; https://doi.org/10.1172/jci.insight.195341DS1). These data suggest that a nutrient-driven component maintains glucose metabolism in *Gc*-ablated mice.

Interestingly, HFD resulted in a significant deterioration of glucose tolerance in male, but not female, WT mice (Supplemental Figure 1, E–P). Therefore, we focused further experiments on HFD-fed male mice. Pyruvate tolerance tests showed a consistently lower glycemia throughout the 2-hour time course in male GcKO mice compared with WT, suggesting a significant reduction in ability to convert pyruvate to glucose in the liver (Figure 1, G and H). Furthermore, insulin tolerance tests showed an improvement in glucose levels within the first 30-minute following insulin administration to GcKO mice compared with WT (Figure 1, I and J). These observations prompted us to conduct an in-depth investigation into the metabolic effects of genetic *Gc* ablation on systemic insulin sensitivity and insulin action.

### GcKO mice showed increased energy expenditure.

**Figure 2:**
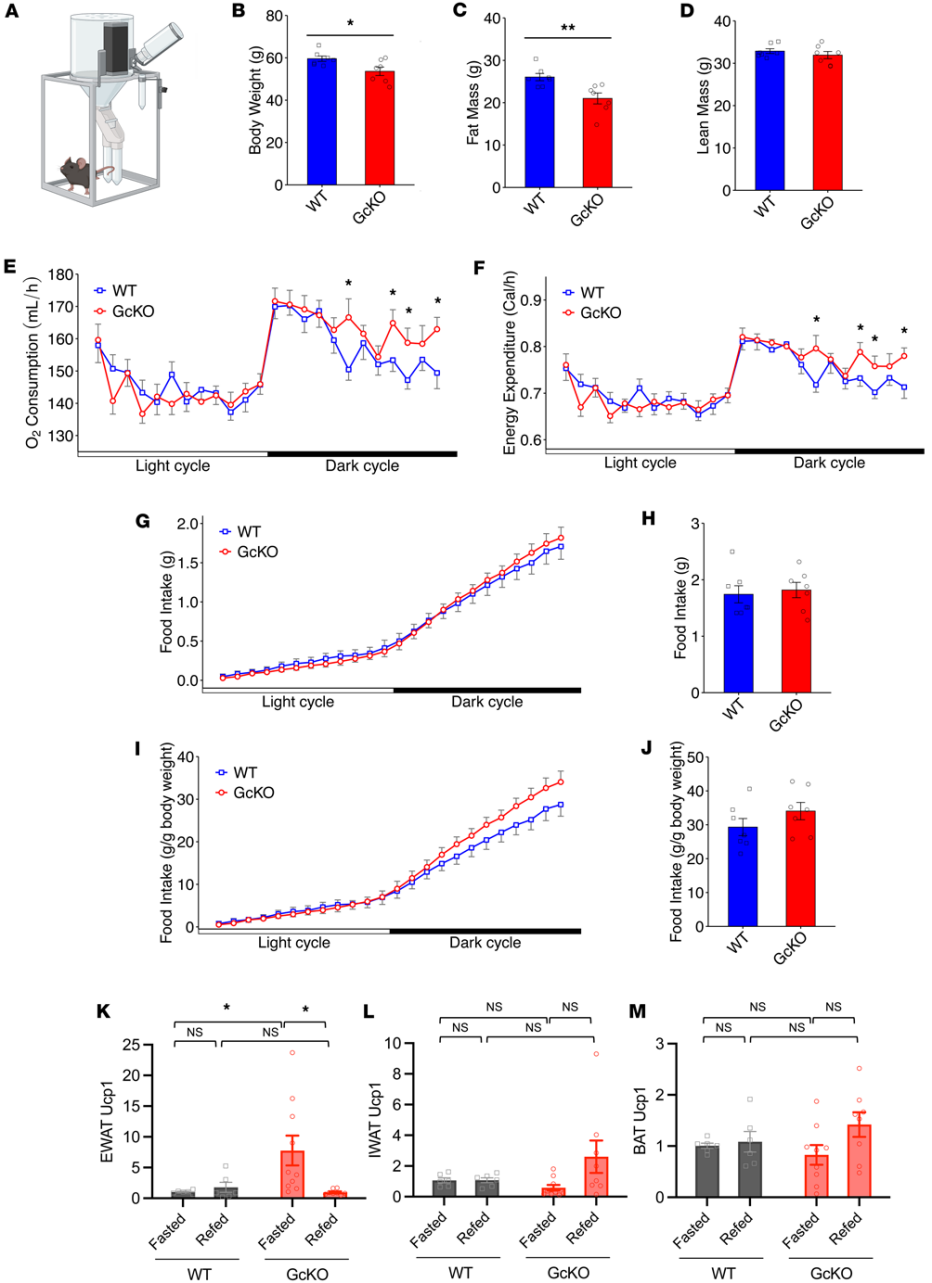
Indirect calorimetry shows increased energy expenditure in the absence of Gc. (**A**) Schematic of mouse metabolic cage used to study energy homeostasis. (**B**–**D**) A group of WT (*n* = 7) and GcKO (*n* = 7) male mice were subjected to indirect calorimetry analysis, where their body weights are shown in **B**, fat mass shown in **C**, and lean mass shown in **D**. (**E**–**J**) Indirect calorimetry revealed oxygen consumption per light and dark cycle (**E**), energy expenditure per light and dark cycle (**F**), food intake per light and dark cycle (**G**) and per day (**H**), and food intake normalized to body weight per light and dark cycle (**I**) and per day (**J**) for WT and GcKO mice in **B**. (**K**–**M**) Ucp1 gene expression in visceral/epidydimal white adipose tissues (EWAT) (**K**), s.c./inguinal white adipose tissues (IWAT) (**L**), and brown adipose tissues (BAT) (**M**). “NS” indicates no significant statistical difference. Data are shown as mean ± SEM, *P < 0.05, **P < 0.01 by Student’s *t* test for **B**, **C**, **D**, **H**, and **J**, by Student’s *t* test for **E**–**G** and **I** at different time points between genotypes, or by 2-way ANOVA for **K**–**M**.

To determine whether *Gc* ablation affected energy homeostasis, we performed indirect calorimetry measurements on HFD-fed WT and GcKO mice (Figure 2A). We first assessed body composition in these mice (Figure 2, B–D). Analyses of indirect calorimetry data found increased oxygen consumption (Figure 2E), carbon dioxide expiration (Supplemental Figure 2A), and energy expenditure (Figure 2F) in GcKO mice, providing a plausible explanation for the reduced body weight compared with WT (Figure 1, A and B, and Figure 2, B and C). Moreover, the increased heat production in GcKO mice did not result from a change in activity (Supplemental Figure 2B). To estimate fat versus carbohydrate utilization, we measured respiratory exchange rate (RER). As both WT and GcKO mice were fed a HFD, their RER was consistently below 0.85, suggesting both groups use fat as the predominant fuel (Supplemental Figure 2C).

To assess the contribution of food intake to reduced body weight in GcKO mice, we measured food consumption stratified by light and dark cycles (Figure 2G) and per day (Figure 2H), and we found no significant difference in food consumption between WT and GcKO. To eliminate the confounding by reduced body weight in GcKO, we further normalized food intake to body weight, but we found no statistically significant difference compared with WT (Figure 2, I and J).

Uncoupling protein 1 (Ucp1) is found primarily in brown adipose tissue and functions to generate heat through nonshivering thermogenesis (30). Another member in the uncoupling protein family, Ucp2, is expressed ubiquitously in various tissues including intestine, lung, spleen, gonadal white adipose tissue (WAT), and brown adipose tissue (BAT), and plays a protective role against atherosclerosis and hypertension (31). To examine if browning in WAT contributes to the observed increase in energy expenditure, we measured Ucp1 mRNA levels in visceral/epidydimal WAT (EWAT) and s.c./inguinal WAT (IWAT), as well as BAT. Compared with WT, fasted GcKO showed increased Ucp1 expression in EWAT (Figure 2K), while no significant difference was observed in IWAT or BAT (Figure 2, L and M). As a control, we also measured Ucp2 mRNA levels. No significant difference was found between WT and GcKO EWAT and IWAT (Supplemental Figure 2, D and E). Moreover, fasted GcKO Ucp2 levels in BAT were reduced compared with refed GcKO, fasted WT, and refed WT (Supplemental Figure 2F). Based on the small but significantly elevated Ucp1 levels in fasted GcKO EWAT, browning may contribute to augmented energy expenditure in GcKO mice.

### GcKO mice showed improved insulin sensitivity.

**Figure 3:**
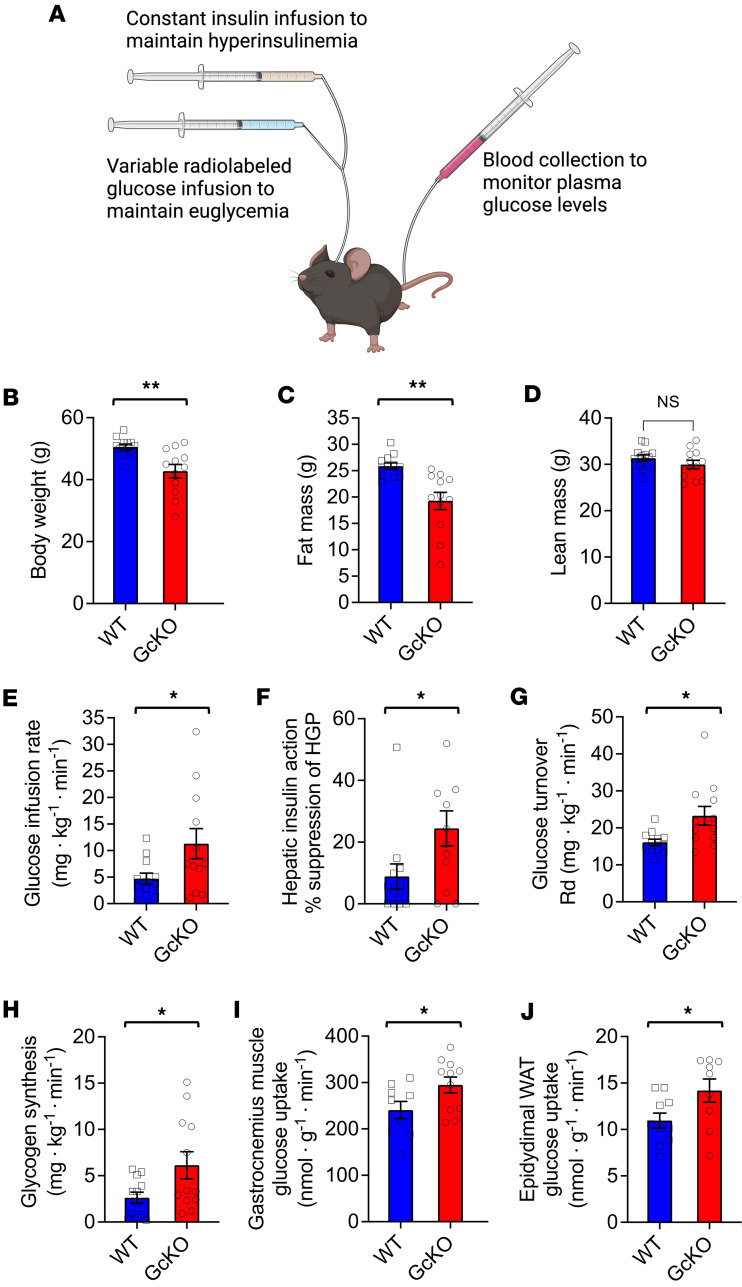
Hyperinsulinemic-euglycemic clamps in WT and GcKO mice. (**A**) Schematic of hyperinsulinemic-euglycemic clamp to measure insulin sensitivity in vivo. (**B**–**D**) Body weight, fat mass, and lean mass of WT and GcKO mice subjected to hyperinsulinemic-euglycemic clamps. (**E**) Glucose infusion rate (GIR) in HFD-fed WT and GcKO mice during clamps. (**F**) Hepatic insulin action, calculated as the ratio of clamp hepatic glucose production (HGP) to basal HGP, and presented as percent suppression. (**G**) Glucose turnover rate, also known as rate of glucose disposal (Rd). (**H**) Whole body glycogen synthesis. (**I**) Glucose uptake in gastrocnemius muscle. (**J**) Glucose uptake in epidydimal fat pad. Mice are males. WT *n* ≥ 12, GcKO *n* ≥ 9. Data are shown as mean ± SEM, *P *<* 0.05, **P < 0.01 by Student’s *t* test.

While the results of ipGTT and ITT (Figure 1) suggest that GcKO mice have improved insulin sensitivity compared with WT, these methods do not eliminate possible confounding by improved insulin secretion or compensatory responses to ITT-induced hypoglycemia, respectively, in GcKO mice (11). We therefore conducted the gold-standard in vivo assessment of insulin sensitivity in HFD-fed WT and GcKO mice: the hyperinsulinemic-euglycemic clamp (Figure 3A) (32). We first performed body composition analysis of WT and GcKO mice (Figure 3, B–D), and we then clamped their glucose levels at ~125 mg/dL using a constant insulin infusion at a rate of 2.5 mU/kg/min and measured the rate of glucose infusion necessary to maintain euglycemia. Compared with WT, GcKO mice need a > 2-fold higher glucose infusion rate to maintain euglycemia (11.3 ± 2.8 versus 4.7 ± 1.0 mg/kg/min; *P* < 0.04) (Figure 3E). Hepatic glucose production (HGP) in GcKO was suppressed by ~25% following insulin clamp, while the minimal response to insulin in WT mice (~9%; *P* < 0.03) indicated a failure to meaningfully respond to insulin (Figure 3F).

Glucose turnover is significantly higher in GcKO mice compared with WT (23.3 ± 2.5 versus 16.1 ± 0.8 mg/kg/min; *P* < 0.02) (Figure 3G). GcKO mice also show increased glycogen synthesis compared with WT (6.1 *±* 1.5 versus 2.6 *±* 0.6 mg/kg/min; *P* < 0.04) (Figure 3H), while glycolysis was similar in both genotypes. Overall, these data indicate that *Gc* ablation is associated with preserved insulin sensitivity following HFD, most notably with regard to insulin’s ability to suppress HGP.

Skeletal muscle is the major site of insulin-stimulated glucose uptake (75%–80%) in humans (33). We accordingly examined glucose uptake in skeletal muscle during hyperinsulinemic-euglycemic clamps and found significantly increased glucose uptake in the gastrocnemius muscles of GcKO compared with WT mice (318 *±* 27 versus 208 *±* 27 nmol/g/min; *P* < 0.03) (Figure 3I). WAT contributes 10%–20% of whole-body glucose uptake in response to insulin (34, 35). Therefore, we also measured glucose uptake in EWAT and found a significant increase in GcKO compared with WT (19 *±* 3.3 versus 10.6 *±* 1.5 nmol/g/min; *P* < 0.05) (Figure 3J). These data demonstrate that, in addition to hepatic insulin sensitivity, *Gc* ablation preserves insulin action on skeletal muscle and s.c. adipose tissue glucose uptake.

### Weight-matched GcKO mice show improved glucose tolerance.

**Figure 4:**
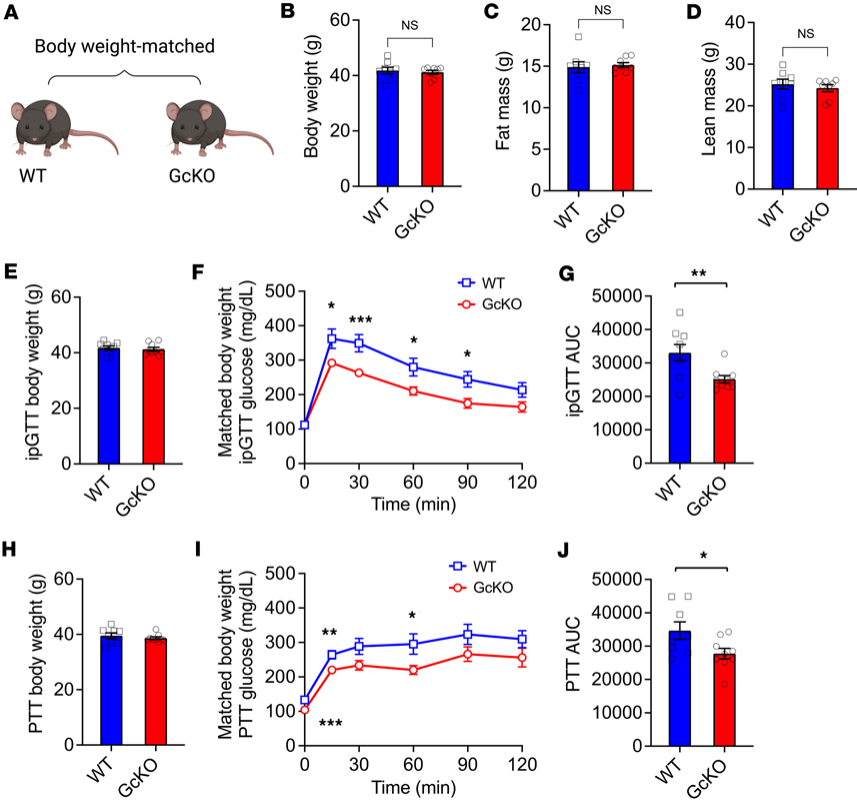
Dissociation of body weight, fat mass, and glucose and pyruvate tolerance in GcKO mice. (**A**) Experimental design with body weight–matched WT and GcKO mice. (**B**–**D**) Body composition from a selected group of WT and GcKO mice with matched body weight, where their body weights are shown in **B**, fat mass shown in **C**, and lean mass shown in **D**. (**E**) Body weights of WT and GcKO mice with matched body weight on the day of glucose tolerance tests. (**F**) Glucose tolerance tests in **D**. (**G**) AUC in **E**. (**H**) Body weights from a selected group of WT and GcKO mice with matching body weights on the day of pyruvate tolerance tests. (**I**) Pyruvate tolerance tests of mice in **G**. (**J**) AUC in **H**. Mice are males. WT *n* ≥ 8, GcKO *n* ≥ 8. Data are shown as mean ± SEM, *P < 0.05, **P < 0.01, ***P < 0.005 by Student’s *t* test.

To examine the role of body weight in improving the metabolic phenotype of GcKO mice, we selected a group of WT and GcKO mice with matched body weight (WT 41.8 versus GcKO 41.2 g) (Figure 4, A and B). Body composition analysis confirmed that weight-matched WT and GcKO mice have comparable fat (Figure 4C) and lean mass (Figure 4D). Next, we performed glucose tolerance tests to evaluate if weight- and fat mass–matched *Gc* KOs would still demonstrate improvement in glucose tolerance. Compared with WT, despite having similar body weights measured again on the day of GTT (Figure 4E), GcKO mice showed increased tolerance to glucose (Figure 4, F and G). We also found improved pyruvate tolerance in GcKO mice with body weight matched to WT (Figure 4, H–J). These data indicate that the improved phenotype in GcKO mice, at least in part, is body weight independent.

### Weight-matched GcKO mice, food intake, and insulin sensitivity.

**Figure 5:**
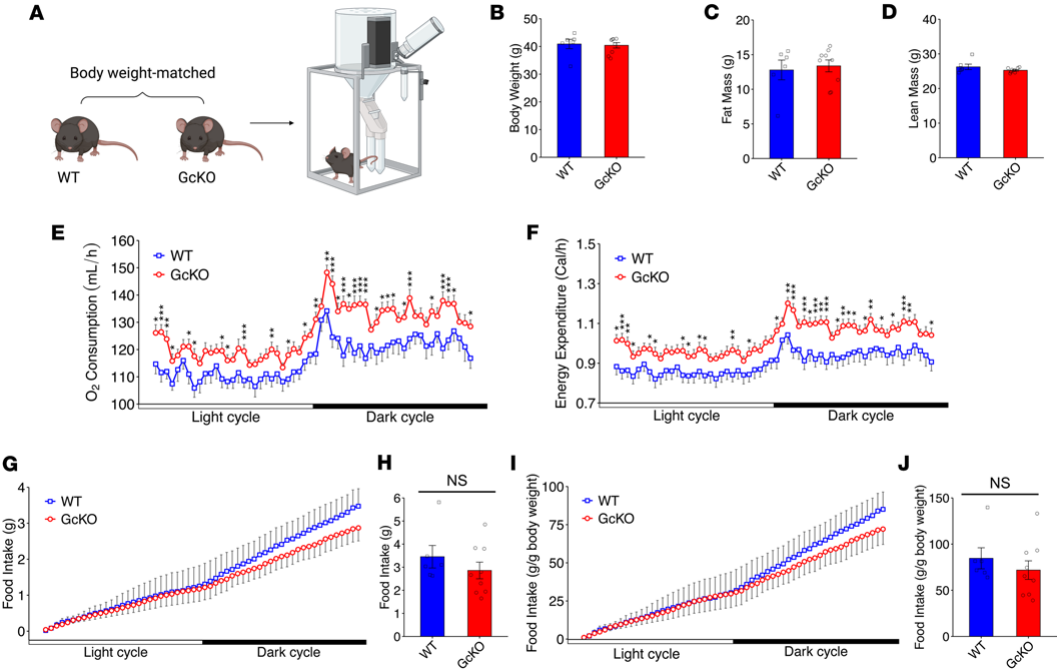
Indirect calorimetry in weight-matched WT and GcKO mice shows increased energy expenditure in the absence of Gc. (**A**) Body weight–matched WT and GcKO mice were subjected to metabolic cages. (**B**–**D**) A group of WT (*n* = 6) and GcKO (*n* = 9) male mice were subjected to indirect calorimetry analysis, where their body weights are shown in **B**, fat mass shown in **C**, and lean mass shown in **D**. (**E**–**J**) Indirect calorimetry revealed oxygen consumption per light and dark cycle (**E**), energy expenditure per light and dark cycle (**F**), food intake per light and dark cycle (**G**) and per day (**H**), and food intake normalized to body weight per light and dark cycle (**I**) and per day (**J**) for WT and GcKO mice in **B**. Data are shown as mean ± SEM, *P < 0.05, **P < 0.01, ***P < 0.005 by Student’s *t* test. For **E**–**G**, and **I**, Student’s *t* test was performed at each time point between genotypes.

To eliminate possible confounding by reduced body weight on energy expenditure, we again used weight- and fat mass–matched WT and GcKO mice, and we subjected them to indirect calorimetry. Body composition analysis again found that body weight–matched (Figure 5, A and B) WT and GcKO mice have comparable fat mass (Figure 5C) and lean mass (Figure 5D).

Weight-matched GcKO mice show increased oxygen consumption (Figure 5E), carbon dioxide expiration (Supplemental Figure 3A), and energy expenditure (Figure 5F). Consistent with our earlier findings, the increased heat production in GcKO mice did not result from an increase in activity (Supplemental Figure 3B). Both WT and GcKO showed a RER below 0.85 and used fat as the predominant fuel (Supplemental Figure 3C).

To assess the contribution of food intake to the reduced body weight of GcKO mice, we measured food consumption per light and dark cycle (Figure 5G) and per day (Figure 5H). We found no difference in food consumption per day between WT and GcKO (Figure 5, G and H). Moreover, when we normalized food intake to body weight, no difference was found between groups (Figure 5, I and J). These data indicate that *Gc* ablation leads to increased energy expenditure and that this effect is independent of body weight.

In order to directly determine insulin sensitivity in vivo, and to account for possible confounding by improved insulin secretion and reduced body weight, we performed hyperinsulinemic-euglycemic clamps in body weight–, fat mass–, and lean mass–matched WT and GcKO mice (Supplemental Figure 4, A–C). Compared with WT, GcKO mice showed significantly improved hepatic insulin action measured by percent suppression of HGP (Supplemental Figure 4D) and glucose turnover rate (Supplemental Figure 4E). Therefore, weight-matched GcKO mice consistently show improved insulin sensitivity compared with WT.

### Improved insulin signaling in GcKO liver.

We next examined whether the preserved insulin sensitivity in GcKO mice was accompanied by changes in gene expression by measuring mRNA levels of several key metabolic genes in the liver, the main site of *Gc* expression (Supplemental Figure 5A). The liver is a complex tissue that performs a plethora of functions with various cell types, including hepatocytes, hepatic stellate cells, Kupffer cells, and endothelial cells. To further explore the expression of Gc, we searched Mouse Liver Atlas (https://www.livercellatlas.org/), which utilized single-cell CITE-Seq to survey mouse liver transcriptomics (36). We found that Gc is highly expressed in the hepatocytes while sparsely in other liver cell types (Supplemental Figure 5, B and C).

qPCR confirmed that *Gc* mRNA was effectively ablated in GcKO mice (Supplemental Figure 5D). Levels of mRNA encoding the gluconeogenic rate-limiting enzyme *Pck1* were decreased in fasted GcKO mice (Supplemental Figure 5E), while expression levels of gluconeogenic genes *G6pase* and *Fbp1* were decreased in both fasted and refed GcKO mice compared with WT mice (Supplemental Figure 5, F and G), consistent with preserved hepatic insulin action found during the euglycemic clamps. In contrast, there was no difference in the glycolytic gene *Gck* (Supplemental Figure 5H) or in the glycogen synthase inhibitor Gsk3β (Supplemental Figure 5I).

To investigate the underlying mechanism of improved insulin sensitivity in HFD-fed GcKO mice, we examined insulin-signaling substrates in the liver. We found increased levels of the insulin signaling mediator Akt phosphorylated at serine 473 (normalized to total Akt) in GcKO mice after refeeding (Supplemental Figure 5, J–M), consistent with augmented hepatic insulin action during hyperinsulinemic-euglycemic clamps (Figure 3E).

Increased levels of plasma nonesterified fatty acid (NEFA) impair insulin’s ability to suppress HGP (37) and can cause insulin resistance (38) as well as hepatic lipid accumulation. We found that NEFA levels were decreased by ~17% in GcKO compared with WT (Supplemental Figure 6A). In addition, plasma triglyceride (TG) levels are generally elevated in those with poorly controlled diabetes as well as in those with prediabetes and are thought to predispose to atherosclerosis (39), and we found that TG levels were ~35% lower in GcKO mice compared with WT (Supplemental Figure 6B). In contrast, we did not find differences in total cholesterol plasma levels (Supplemental Figure 6C).

To assess whether the decreased levels of NEFA and TG are a consequence of decreased circulating glucagon during the fasted state, we measured plasma glucagon levels in WT and GcKO mice. While refeeding lowered glucagon secretion in both WT and GcKO, we did not find differences in fasted or refed state between genotypes (Supplemental Figure 6D). These data indicate that *Gc* ablation did not alter glucagon secretion and that the changes in lipid profiles in GcKO mice are not related to glucagon levels. While there was no difference in hepatic TG levels (Supplemental Figure 6E), we found a ~50% reduction in hepatic glycogen content in GcKO compared with WT (Supplemental Figure 6F), consistent with increased insulin action in skeletal muscle and adipose tissue. These data highlight the contribution of the liver to improved insulin signaling in GcKO mice, and they focus our genome-wide RNA profiling effort to the liver.

### RNA-Seq data reveal altered signaling and pathways in the absence of Gc.

**Figure 6:**
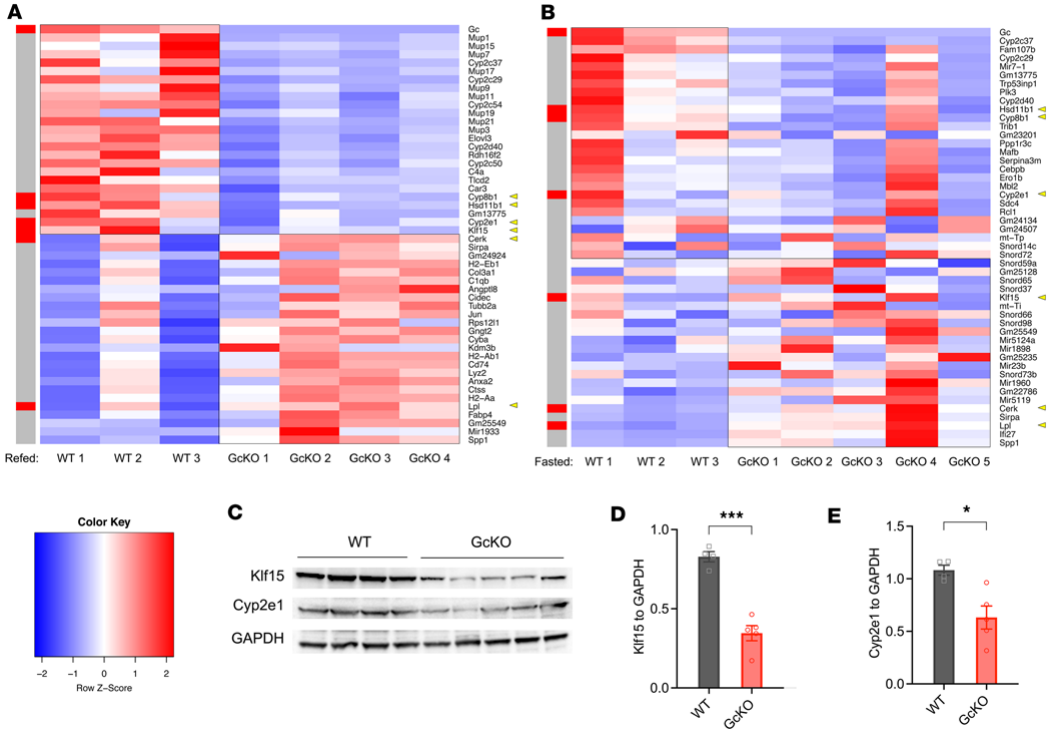
Heatmaps and immunoblotting of WT and GcKO liver. (**A** and **B**) HFD-fed WT and GcKO male mice were fasting for 16 hours (fasted condition), or fasted for 16 hours followed by 2 hours refeeding (refed condition), and subjected for RNA-Seq. Heatmaps represent top differentially expressed genes between refed WT (*n* = 3) versus refed GcKO (*n* = 4) (**A**) and fasted WT (*n* = 3) versus fasted GcKO (*n* = 5) mice (**A**), with TPM ≥ 1. The gray bar on the left highlighted genes of interest in red. (**C**) Immunoblotting in refed WT and GcKO liver with antibodies against Klf15, Cyp2e1, and GAPDH (internal control). (**D**) Quantification for Klf15 normalized to GAPDH in **C**. (**E**) Quantification of Cyp2e1 normalized to GAPDH in **C**. Data are shown as mean ± SEM, *P < 0.05, ***P < 0.005 by Student’s *t* test.

We performed RNA-Seq on liver extracts from HFD-fed, fasted, and refed WT and GcKO mice. We applied stringent thresholds of *P* < 0.05 and *P*_adj_ < 0.1, and we obtained 878 differentially regulated genes (DRGs) comparing GcKO to WT under the refed (2-hour) condition (Figure 6A, Supplemental Figure 7A, and Supplemental Datasets 1 and 2) and 968 DRGs under the fasted (16-hour) condition (Figure 6B, Supplemental Figure 7B, and Supplemental Datasets 3 and 4), followed by gene ontology analysis using Ingenuity Pathway Analysis (Qiagen) (Tables 1 and 2). Table 1 shows gene ontologies enriched in genes differentially regulated in WT versus GcKO under the refed condition, while Table 2 shows these ontologies in WT versus GcKO under the fasted condition.

Comparing liver DRGs in WT and GcKO (Tables 1 and 2), LPS/IL-1–mediated inhibition of Rxr function and Nrf2-mediated oxidative stress response pathways are common pathways to both fasted and refed conditions. Next, Acox1 and Ppara are top upstream regulators unique to the refed condition (Table 1), while Tead1 is unique to the fasted condition (Table 2). In contrast, dexamethasone and LPS are top upstream regulators common to both fasted and refed conditions. Furthermore, fatty acid metabolism is common among the top toxic lists in both conditions, and altered lipid metabolism is common among the top molecular and cellular function lists (Tables 1 and 2). In contrast, altered amino acid metabolism is unique to the refed condition (Table 1), while altered carbohydrate metabolism is unique to the fasted condition (Table 2).

Refed GcKO mice showed reduced *Cyp8b1* expression in the liver compared with WT. Interestingly, *Cyp8b1*-KO mice were resistant to Western diet–induced body weight gain, hepatic steatosis, and insulin resistance (40) (Figure 6A and Supplemental Figure 7A). Moreover, we found lower levels of *Hsd11b1*, which encodes the enzyme that catalyzes the interconversion of inactive cortisone to active cortisol. Increased *HSD11B1* is associated with metabolic disorders in patients with obesity (41) (Figure 6A and Supplemental Figure 7A). Similarly, GcKO mice showed reduced levels of *Cyp2e1*, which encodes an enzyme increased in nonalcoholic fatty liver disease (NAFLD) and nonalcoholic steatohepatitis (NASH) (42) in the liver (Supplemental Figure 7A). KLF15 plays a role in negatively regulating HGP after refeeding (43), and ablation of Gc reduced hepatic *Klf15* levels (Figure 6A and Supplemental Figure 7A). Furthermore, CERK positively regulates the protein expression of NRF2, which is crucial to maintaining redox homeostasis (44). Our data suggest that deletion of Gc increases *Cerk* to help maintain redox homeostasis in the liver under metabolic challenges (Figure 6A and Supplemental Figure 7A). Liver-specific overexpression of lipoprotein lipase improves glucose metabolism in HFD-fed mice (45). Here, we found that GcKO mice showed increased *Lpl* in the liver, consistent with the improved glucose tolerance in GcKO mice (Figure 6A and Supplemental Figure 7A). These expression changes in the liver can help explain the improved metabolic profile in GcKO mice.

Based on the above gene ontology analysis focused on liver-related genes, we hypothesize that changes in oxidative stress and gluconeogenic pathways are mechanisms underlying the observed improved metabolic phenotypes in GcKO mice. To test this hypothesis, we performed Western blotting to measure protein levels of Klf15 and Cyp2e1 (Figure 6C). It has been shown that acute depletion of hepatic Klf15 in mouse results in reduced mRNA levels of gluconeogenic genes Pck and G6pc and improves glucose tolerance in db/db mice compared with WT (43). These improved metabolic phenotypes in Klf15-knockdown mice are consistent with those of GcKO mice (Figure 1, E–H, and Supplemental Figure 5, C and D). Interestingly, we found that GcKO mice showed a ~60% reduction of hepatic Klf15 protein levels (Figure 6D). These results suggest that Klf15 could mediate the effect of Gc in the liver.

Hepatic CYP2E1 is increased in murine models of steatohepatitis and in patients with NASH (46). While hepatic cytochrome P450s, such as Cyp2e1, play central roles in metabolizing toxic xenobiotics, superoxide anions and hydrogen peroxide produced during this process contribute to hepatic oxidative stress (47). Chronic hepatic oxidative stress can lead to mitochondrial dysfunction, liver injury, and metabolic disorders. To this end, we assessed Cyp2e1 protein levels in WT and GcKO liver, and we found that, in GcKO liver, Cyp2e1 protein levels are reduced compared with WT (Figure 6E). These results suggest that deletion of Gc could have a protective effect against hepatic oxidative stress.

### Acute lowering of Gc levels improves glucose tolerance in HFD-fed mice.

**Figure 7:**
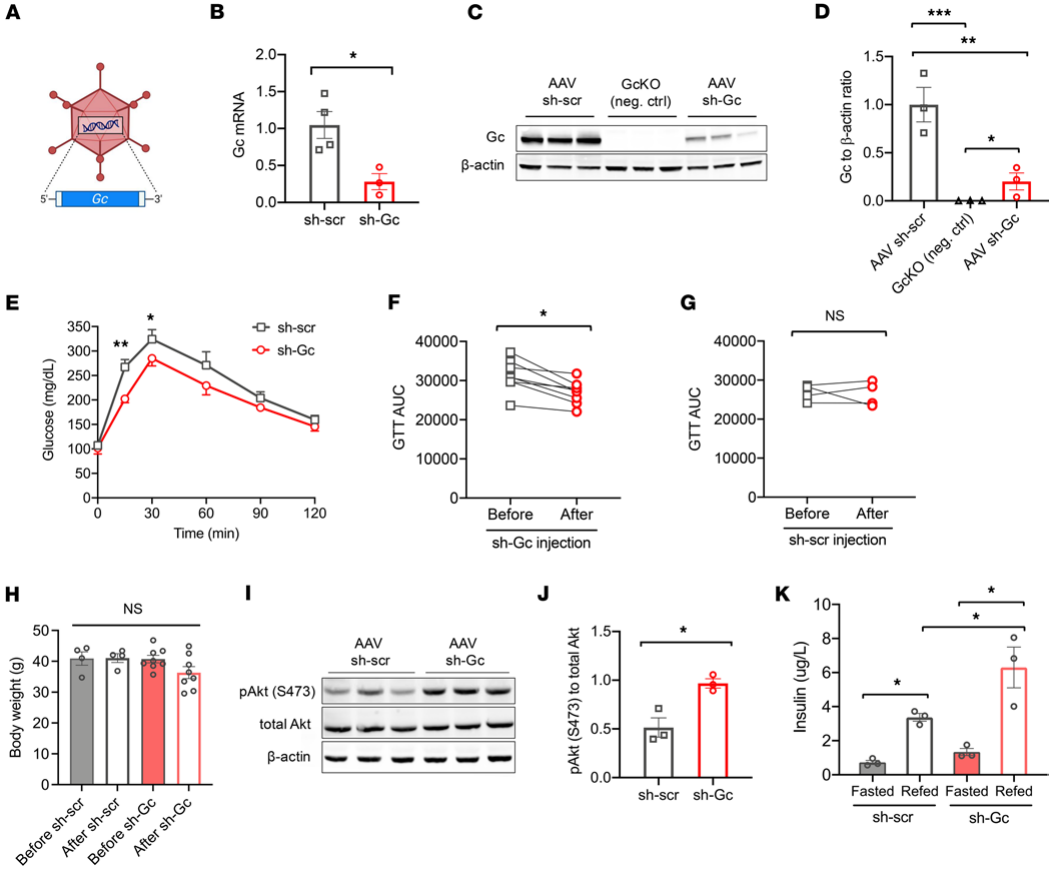
Inhibition of hepatic Gc with adeno-associated virus (AAV). (**A**) Schematic of adeno-associated virus (AAV) targeting *Gc* in vivo. (**B**) *Gc* expression in WT mice injected with AAV encoding shRNA against control (sh-scr) or *Gc* (sh-Gc). (**C**) Immunoblotting of liver Gc and internal control β-actin in WT mice injected with AAV carrying sh-scr or sh-Gc. GcKO mice were used as a negative control. (**D**) Quantification of **B**. (**E**) Glucose tolerance tests in WT mice injected with control (sh-scr, *n* = 4) or *Gc* (sh-Gc, *n* = 8) shRNA. (**F**) AUC from glucose tolerance tests in individual WT mice before and after AAV sh-Gc injection (*n* = 8). (**G**) AUC from glucose tolerance tests in individual WT mice before and after AAV sh-scr injection (*n* = 4). (**H**) Body weight of mice before and after sh-scr (*n* = 4) or sh-Gc (*n* = 8) AAV injection. (**I**) Immunoblotting of insulin signaling substrates in the liver of mice receiving sh-scr or sh-Gc AAV injection. (**J**) Quantification of **H**. (**K**) Plasma insulin levels in sh-scr and sh-Gc mice in fasted and refed conditions. Data are shown as mean ± SEM, *P < 0.05, **P < 0.01, ***P < 0.005 by Student’s *t* test in **B**, **E**–**G**, and **J**, 1-way ANOVA in **D**, or 2-way ANOVA in **H** and **K**.

The changes observed in GcKO mice may reflect adaptive changes to chronic *Gc* deficiency. To investigate whether acute inhibition of Gc levels can improve insulin sensitivity, we injected adeno-associated virus (AAV8) carrying shRNA against *Gc* (sh-Gc) (Figure 7A) to inhibit *Gc* production in the liver of HFD-fed mice, compared with control (sh-scr). Expression analyses confirmed that sh-Gc decreased *Gc* mRNA (Figure 7B) and protein levels (Figure 7, C and D) by ~70% in whole liver. Three weeks after injection, we performed glucose tolerance tests and found a significant improvement in glucose tolerance in sh-Gc mice compared with sh-scr (Figure 7E). Analyses of individual mice before and after AAV injection indicate that the decrease occurred across the board in virtually all mice examined (Figure 7F). In contrast, no significant difference was observed in sh-scr–injected mice (Figure 7G). Moreover, this improvement of glucose tolerance was not accompanied by weight loss (Figure 7H). This result suggests that the beneficial effects in *Gc*-null mice have body weight–dependent and body weight–independent components. Additionally, we observed elevated hepatic phosphorylated Akt levels in mice receiving sh-Gc injection (Figure 7, I and J). Interestingly, acute ablation of *Gc* also improved plasma insulin levels after refeeding (Figure 7K).

## Discussion

Previously, we reported that *Gc* is ectopically activated in β cells in diet-induced obesity and contributes to β cell dedifferentiation and dysfunction. Ablation of *Gc* resulted in increased insulin secretion in hyperglycemic clamps in HFD-fed animals and in isolated islets, which indicate a role of *Gc* in diabetic pancreatic β cell failure (11). The main findings of the current work are: (a) *Gc* ablation in mice reduced fat mass and body weight following HFD, and it lowered fasting glycemia, plasma NEFA, and TG levels; (b) *Gc*-deficient mice also showed improved insulin sensitivity in liver, adipose tissue, and skeletal muscle and improved glucose and pyruvate tolerance; (c) there were no alterations in food intake in *Gc*-deficient mice; (d) acute inhibition of *Gc*, achieved using adeno-associated virus to target hepatic *Gc* mRNA, improved glucose tolerance in HFD-fed mice; and (e) improvements in hepatic oxidative stress and gluconeogenic pathways provide an explanation for the augmented insulin sensitivity in the Gc-ablated liver. The present data add a potentially new and unforeseen dimension to the gamut of Gc functions by demonstrating that its ablation preserves insulin sensitivity when mice are fed a metabolically unhealthy diet. Thus, Gc inhibition has a dual effect to maintain β cell insulin secretion and insulin sensitivity. The weight reduction in *Gc*-ablated mice did not result from appetite suppression, suggesting that the effects occur primarily through a brain-independent mechanism. Moreover, the weight loss in the absence of *Gc* resulted from fat mass reduction and spared muscle mass. Finally, adding to the potential translational interest of these observations is the fact that Gc can be targeted either as a circulating factor or through the liver, where it is primarily expressed.

Gc is primarily a vitamin D binding protein (48). About ~85% of circulating 25-hydroxyvitamin D [25(OH)D] is bound to Gc, with the remainder weakly bound to albumin, from which it becomes dissociated in order to enter tissues. Only ~0.03% of 25(OH)D circulates freely in blood and has direct access to target cells. Both the free and albumin-bound 25(OH)D fractions are viewed as bioavailable and engage in their metabolic functions (49). In contrast, Gc-bound vitamin D and its metabolites are biologically unavailable, with the exception of tissues such as the kidney that express the megalin/cubilin transport system, which allows for Gc-bound 25(OH)D to enter cells (50, 51). The half-life of circulating Gc is 2.5–3 days. Gc possesses a single binding site for all vitamin D metabolites, including the active form of vitamin D — 1,25 (OH)_2_D — and the parental form of vitamin D, with highest affinity for the latter and major form of plasma vitamin D — 25(OH)D (48). Although Gc-bound 25(OH)D is mostly biologically unavailable, Gc acts as a reservoir for circulating 25(OH)D by increasing its half-life, as 25(OH)D is rapidly metabolized and excreted in the urine in the absence of Gc (7). While total concentrations of 25(OH)D and 1,25(OH)_2_D in *Gc*-deficient mice (7) and in nullizygous *GC* humans (28, 29) are extremely low, calcium and bone homeostasis remain relatively normal. Therefore, *Gc*-null mice only showed a mild bone mineralization defect when placed on a vitamin D–deficient diet for 4 weeks (7). Furthermore, Gc plays a role in transporting fatty acids (52). These findings are consistent with the possibility that there are other functions of Gc, in addition to transporting vitamin D.

The relationship between diabetes and vitamin D levels has long been debated. Several observational and physiological studies have linked vitamin D insufficiency with β cell dysfunction, insulin resistance, and obesity, both in adults and children (53). However, the pivotal D2d clinical trial, a randomized control trial in individuals at risk of developing diabetes, showed no statistically significant benefit from vitamin D supplementation (54), nor did similar trials (55, 56). Thus, the balance of probabilities is that the effect of *Gc* deficiency is independent of vitamin D levels, especially since the latter tend to be low in the plasma when *Gc* is ablated. This point was further demonstrated by the identification of a patient homozygous for *GC* loss-of-function mutations (29). Although this patient has undetectable GC protein with very low 25(OH)D and 1,25(OH)D levels, they had no apparent bone abnormalities.

If what we currently know about Gc’s primary function as a vitamin D binding protein is not the whole story, what additional function can Gc possess? A report on plasma proteome association with human diseases and traits provides a few insights (57). A total of 660 human diseases were analyzed for its association with GC protein (https://proteome-phenome-atlas.com/) (57). Among them, GC protein is the most significantly and positively associated with disorders of lipoprotein metabolism in endocrine disorders and digestive system diseases (*P* < 0.01) (Supplemental Table 1). These associations provide further clues to deciphering the function of GC protein in metabolism.

Case reports of 2 individuals who are homozygous null for mutations in *GC* confirmed its role as a vitamin D carrier (28, 29). The first patient was a 58-year-old woman who carried two 139 kb deletions spanning the gene body of *GC* as well as 133 kb deletions eliminating part of another gene, neuropeptide FF receptor 2 (*NPFFR2*). She had a history of musculoskeletal pain, fragility fractures, and ankylosing spondylitis as well as extremely low plasma levels of vitamin D and its metabolites. Although she had diabetes, it was well controlled on metformin (J. Marcadier, personal communication; University of Calgary, Calgary, Alberta, Canada) and was associated with additional comorbidities, including hypertension, dyslipidemia, NAFLD, and autoimmunity, that may have metabolic effects of their own (28). The second patient was a 60-year-old man homozygous for pathogenic variants affecting a canonical splice site in *GC* and had undetectable to low levels of 25(OH)D and 1,25(OH)_2_D (29). He otherwise did not phenocopy the first patient, suggesting the first patient’s fragility fractures were attributable to her rheumatic disease rather than vitamin D deficiency. Additional clinical differences may be due to other background genetic differences, possibly including those within or near the *NPFFR2* deletion. It should be emphasized that our experiments show beneficial effects of ~70% Gc inhibition in mice and, together with the case reports of viable *GC* null individuals, suggest that the therapeutic window of inhibition necessary for metabolic benefits can be safe in humans.

We are left with the question of how *Gc* deficiency promotes insulin sensitivity. There are precedents for circulating proteins that modulate insulin action — for example, adiponectin (58), Resistin (59), and Rbp4 (60). The beneficial effects of *Gc* ablation in HFD-fed GcKO mice run the gamut from decreased weight and fat mass to decreased HGP, NEFA, and TG and increased insulin action on glucose transport. This combination of actions is most consistent with a systemic insulin-like property associated with *Gc* ablation. One possibility is that Gc carries 1 or more biochemical entities that confer insulin resistance; thus, its ablation confers protection against this agent(s). Alternatively, Gc can act to prop up insulin receptor signaling, either by acting at the cell membrane or through some heretofore unrecognized intracellular mechanism. However, the latter would require that Gc be taken up by cells, and this property appears to be limited to the kidney. More work will be required to address these questions.

Single-cell CITE-Seq in mouse liver revealed that hepatocytes express the highest level of Gc among liver cell types (36), which suggests an involvement of hepatocytes in conferring the improved metabolic phenotypes in the absence of Gc. Moreover, adeno-associated virus serotype 8 (AAV8) was chosen for the acute Gc-KO experiment for its strong affinity and high efficiency in targeting hepatocytes (61). However, determination of whether the observed phenotypes resulted from hepatocyte cell autonomous actions and/or paracrine effects between liver cell types will require further investigation.

From a therapeutic standpoint, there remains an unmet need for a single drug acting as insulin secretagogue, insulin sensitizer (62), and fat mass–reducing agent, without appetite suppression or muscle loss. Thiazolidinediones, whose use has fallen out of fashion due to a combination of adverse side effects and regulatory setbacks (63), remain the only class of drugs consistently associated with an effect on diabetes prevention (64–66). They also show consistently lower secondary failure rates than metformin, sulfonylureas (67), and GLP1-RA (68). In addition to insulin sensitization, *Gc* inhibition appears to also protect against β cell dysfunction and increase insulin secretion. Adipose tissue browning can dissipate energy through Ucp1 and has garnered attention as a way to rid of excess energy (69), suggesting that the human body has built-in capacities to eliminate excess calories. We observed a small but significant elevation of Ucp1 mRNA in fasted GcKO EWAT; however, this magnitude of UCP1 mRNA elevation and associated potential browning/beigeing in EWAT, if any, is unlikely to be sufficient to explain the increased whole-body energy expenditure in GcKO mice. Future directions will include investigating Ucp-1–independent thermogenesis processes, such as calcium cycling, creatine-substrate cycling, and lipid cycling (70, 71), to provide mechanistic insights into elevated energy expenditure in the absence of Gc. There is a need for therapeutics that can address β cell function, peripheral insulin action, and fat mass but not muscle mass reduction, without brain effects; we propose Gc as a candidate target.

## Methods

### Sex as a biological variable.

We utilized both male and female mice in our study, and found a robust metabolic phenotype in male but not female mice. This observation is consistent with the understanding in the metabolic field that female mice are more resistant to HFD challenges compared with male mice (72–74).

### Animal care and use.

We generated GcKO using frozen sperm from the UC Davis Knockout Mouse Project Repository [Gc^tm1.1(KOMP)Vlcg^] (11). Mice were weaned at 3 weeks of age, housed at 22°C–24°C, fed normal chow diet (NCD) or HFD, and maintained on a 12-hour light-dark cycle (lights on at 7 AM). Based on caloric content, NCD consists of 13% fat, 62% carbohydrates, and 25% protein (PicoLab rodent diet 20, 5053; Purina Mills), while HFD contains 60% fat, 24% carbohydrates, and 16% protein (Research Diets, D12492). Littermate control mice for GcKO retained 2 WT *Gc* alleles. These mice were maintained on a mixed C57BL/6N-129J genetic background, with the majority being C57BL/6N.

### Metabolic analyses and indirect calorimetry.

We performed i.p. glucose tolerance tests (ipGTT) by injecting glucose (2 g/kg for NCD-fed mice, or 1g/kg for HFD fed mice) after an overnight fast. Insulin tolerance tests were performed by injecting insulin (0.75 units/kg) after a 4-hour fast, and i.p. pyruvate tolerance tests by injecting sodium pyruvate (2g/kg) after an overnight fast (75). We measured insulin levels with an ELISA kit (Mercodia) and glucagon levels with a radioimmunoassay kit (MilliporeSigma). We estimated body composition by nuclear magnetic resonance (Bruker Optics). We measured food intake, energy expenditure, and RER with a Comprehensive Laboratory Animal Monitoring System (CLAMS, Oxymax) (Columbus Instruments). We adapted the CalR calculation model for indirect calorimetry experiments (76). Plasma TG, total cholesterol, and NEFA were measured with commercially available kits following manufacturers’ instructions: Infinity TG kit (Thermo Fisher Scientific), total cholesterol E kit (Wako Diagnostics), and NEFA kit (Wako Diagnostics).

### RNA preparation, qPCR, and RNA-Seq.

We isolated total RNA from whole liver with Nucleospin RNA kit (Macherey-Nagel) with DNase I treatment and followed a previously described protocol for reverse transcription (77). We used the resulting cDNA to perform quantitative PCR with GoTaq master mix (Promega) and analyzed data with the standard ΔΔCt method. Rpl19 was used for internal normalization. Primer sequences are listed in Supplemental Table 2.

Directional Poly-A RNA-Seq libraries were prepared and sequenced as PE100 (100bp paired-end reads) on Illumina NextSeq 500 to a read depth of 40 million read-pairs per sample. The reads were mapped to the mouse genome mm10 using the STAR (v2.5.2b) (78) algorithm with default settings. FeatureCounts from the Subread (v1.5.2) (79) package was used to assign concordantly aligned reads pairs to RefSeq genes. A 25 bp minimum as overlapping bases in a fragment was specified for read assignment. Raw read counts were used as input for DESeq2 (v1.14.1) (80), which was further used to filter out genes with low counts, normalize the counts using library sizes, and perform statistical tests to find differentially expressed genes. For statistical analysis, a standard cutoff of *P* < 0.05 and FDR < 0.1 was applied. Differential expression analysis was conducted for fasted WT versus fasted GcKO, and refed WT versus refed GcKO. Gene ontology and pathway analyses were performed using Ingenuity Pathway Analysis software (Qiagen). Raw and processed data were deposited into the MINSEQE-compliant National Center for Biotechnology Information Gene Expression Omnibus database (GSE288309).

### Western blotting and imaging.

We used a previously described protocol for immunoblotting (81). A 30mg liver sample was placed in 600 µL RIPA lysis buffer (Millipore, 20-188), and supplemented with Halt protease and phosphatase inhibitor cocktail (Thermo Scientific, 78440). The sample was homogenized for 30 seconds, on setting 6 of an IKA T10 Basic Ultra-Turrax homogenizer system. Then, samples were spun down using a benchtop centrifuge at 4°C with 20,817*g* for 10 minutes, and supernatant was transferred to a new tube avoiding the lipid layer and precipitant. The supernatant was sonicated for a total of 5 minutes (10 seconds on and 10 seconds off for 10 minutes) on setting 9 of a model 550 Sonic Dismembrator (Fisher Scientific). The supernatant was spun again and transferred to a new tube avoiding precipitant, followed by protein concentration measurement (Pierce BCA protein assay kit).

The following primary antibodies were used: anti-Gc (HPA019855, Atlas Antibodies, 1:1000 dilution); anti-total Akt (Cell Signaling, no. 9272, 1:1000 dilution); anti-phosphorylated Akt at serine 473 (Cell Signaling, no. 4060, 1:500 dilution); and anti-β actin (Abcam, ab8227, 1:1000 dilution). IRDye 800CW (LI-COR, no. 925-32211) or IRDye 680LT (LI-COR, no. 925-68021) goat anti-rabbit secondary antibody was used. Proteins were detected using an Odyssey imaging system (LI-COR). Furthermore, to validate RNA-Seq results, the following primary antibodies were used: anti-Klf15 (Proteintech no. 13749-1-AP, 1:1000 dilution); anti-Cyp2e1 (Proteintech no. 19937-1-AP, 1:1000 dilution); and anti-GAPDH (Abcam ab9485, 1:1000 dilution). Secondary antibody against rabbit IgG conjugated with peroxidase produced in goat (Sigma A0545, 1:10,000 dilution) was applied for 1 hour, followed by ECL prime western blotting detection reagents (Cytiva Amersham). Bio-Rad ChemiDoc imaging system was used to capture the results. Quantifications were measured using ImageJ.

### Hyperinsulinemic-euglycemic clamps.

We performed hyperinsulinemic-euglycemic clamps as previously described (32). Briefly, during the 120-minute clamp period, insulin was infused at a constant rate of 2.5 mU/kg/min to raise circulating insulin levels about 3-fold over basal levels. Concurrently, glucose was infused i.v. at variable rates to maintain euglycemia, and this rate of glucose infusion directly correlates with insulin sensitivity. Plasma glucose concentrations were measured every 10–20 minutes from the cut tail vein to adjust the rates of glucose infusion needed to maintain a constant glycemia. Furthermore, radioactively labeled [^3-3^H] glucose (18 µCi per mouse) and 2-deoxy-D-[1-^14^C] glucose (a bolus of 10 µCi per mouse) were used to measure HGP and glucose metabolism in skeletal muscle and adipose tissues, respectively.

Prior to the 120-minute clamp period, [^3-3^H] glucose (0.05 µCi/min) was infused for 120 minutes to examine the basal rate of whole-body glucose turnover. Plasma samples were taken at the end of this infusion period to measure basal levels of insulin, plasma glucose, [^3^H] glucose, specific activity, and rates of glucose turnover. During this basal state, HGP is the only source of glucose introduction into the circulatory system, and therefore represents a steady state for peripheral glucose disposal. As a result, the basal rate of whole-body glucose turnover equilibrates with the basal rate of HGP.

During the 120-minute clamp period, [^3-3^H] glucose (0.1 µCi/min) was continuously infused to measure insulin-stimulated whole-body glucose turnover rate. Plasma [^3^H] glucose concentrations recorded during the final 30 minutes of the clamp were used to calculate the specific activity and glucose turnover rates. Furthermore, a one-time, nonmetabolizable 2-deoxy-D-[1-^14^C] glucose infusion at 75 minutes into the clamp period was used to measure glucose uptake in the gastrocnemius muscle and EWAT.

Whole-body glycolysis was obtained from the rate of elevated plasma ^3^H2O as a byproduct of glycolysis, which was calculated using linear regression based on measurements from 5-minute intervals between 80 and 120 minutes. Whole body glycogen synthesis was estimated by subtracting whole-body glycolysis from whole-body glucose turnover. At the end of the clamp period, mice were anesthetized using sodium pentobarbital, and tissues were harvested for biochemical analysis.

### Adeno-associated virus injection.

Because of liver tropism associated with AAV8, AAV8-H1-shRNA was chosen as a vector to mediate the knockdown of hepatic *Gc* (82). We generated AAV8-H1 shRNA against *Gc* (sh-Gc) to silence liver *Gc*, as well as a scrambled shRNA control (sh-scr). Three-month-old WT mice were fed a HFD for 12 weeks prior to viral injection. Viral injection was performed through the tail vein at a dose of 1.5 × 10^11^ genome copies per mouse. Glucose tolerance tests were performed prior to and 3 weeks after viral injection.

### Statistics.

Data were analyzed with Prism 8 (GraphPad) and R 4.5.0 using the ggplot2 4.0.0 (83) and gplots 3.2.0 (84) packages. Results are shown as mean ± SEM. *P* values 0.05 were considered statistically significant. Statistical analyses were carried out using 2-tailed Student’s *t* test when 2 groups were compared, and using ANOVA specified in each figure legend, followed by Turkey’s multiple-comparison test when more than 2 groups are compared.

### Data availability.

Raw and processed RNA-Seq data were deposited into the MINSEQE-compliant National Center for Biotechnology Information Gene Expression Omnibus database (GSE288309). Underlying data are available in the Supporting Data Values file. Datasets generated and/or analyzed during the current study are also available from the corresponding author upon reasonable request.

### Study approval.

UC Davis and Columbia University IACUC approved all animal procedures.

## Author contributions

RG designed the studies, performed experiments, analyzed the data, and wrote the manuscript. TK designed the studies, performed experiments, analyzed the data, and wrote the manuscript.

## Funding support

This work is the result of NIH funding, in whole or in part, and is subject to the NIH Public Access Policy. Through acceptance of this federal funding, the NIH has been given a right to make the work publicly available in PubMed Central.

- UC Davis start-up fund (to TK)
- NIDDK pilot and feasibility grants P30DK98722, P30DK63608, and P30DK116074 (to TK)
- NIH grants K01DK114372 (to TK)

## Supplementary Material

Supplemental data

Supplemental data sets 1-4

Unedited blot and gel images

Supporting data values

## Figures and Tables

**Table 1:**
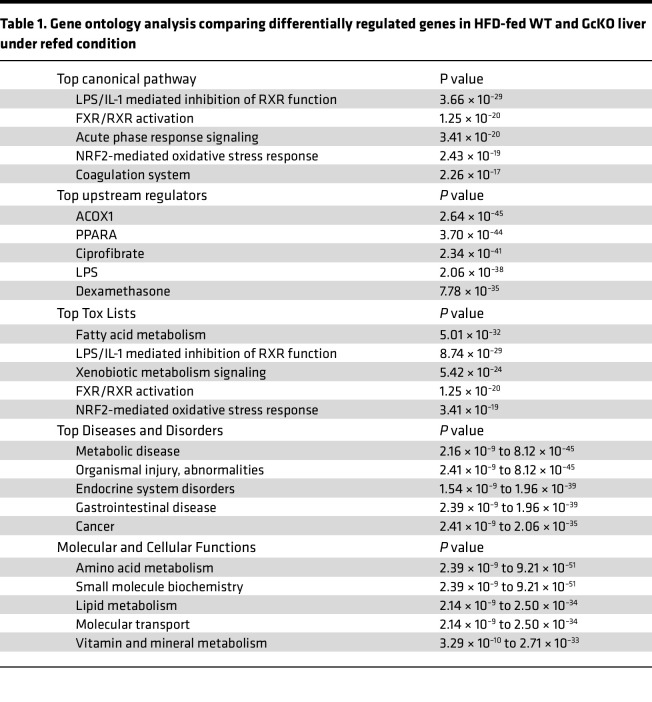
Gene ontology analysis comparing differentially regulated genes in HFD-fed WT and GcKO liver under refed condition

**Table 2:**
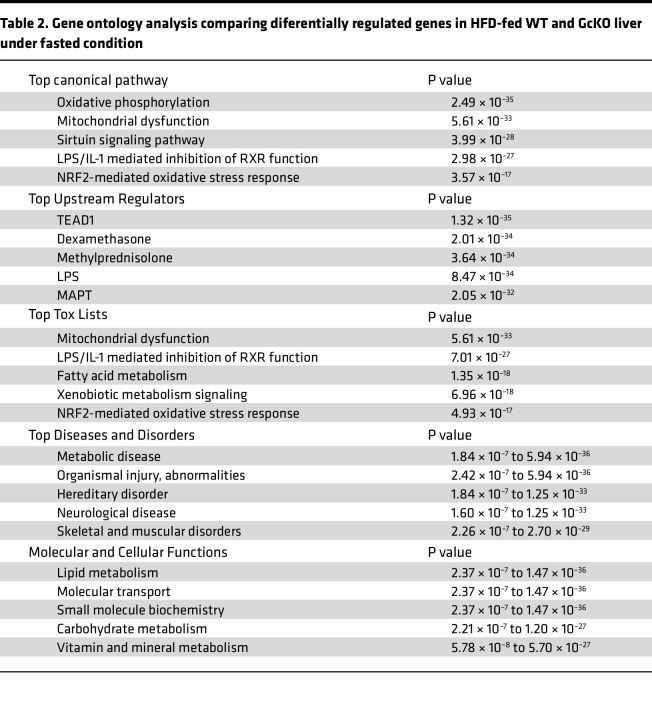
Gene ontology analysis comparing diferentially regulated genes in HFD-fed WT and GcKO liver under fasted condition

## Version 1 12/08/2025

Electronic publication
