# Supplementary material for: Gc inhibition preserves insulin sensitivity and reduces body weight without loss of muscle mass
Source: JCI Insight. 2025 Dec 8;10(23):e195341. doi: 10.1172/jci.insight.195341 (PMC12890482; doi:10.1172/jci.insight.195341)
Supplement: Supplemental data [file jciinsight-10-195341-s249.pdf]

Gc inhibition preserves insulin sensitivity and reduces body weight without loss of muscle mass

Richard Gill[1], Taiyi Kuo[1]

[1]Department of Neurobiology, Physiology, and Behavior, University of California Davis, Davis, USA.

Authors:
Richard Gill, PhD
Department of Neurobiology, Physiology, and Behavior, University of California Davis,
605 Hutchinson Drive, 1111 Green Hall, Davis, CA 95616, USA.
Email: rtgill@ucdavis.edu
ORCID: 0000-0001-7057-7807

Correspondence:
Taiyi Kuo, PhD
Department of Neurobiology, Physiology, and Behavior, University of California Davis,
605 Hutchinson Drive, 1009 Green Hall, Davis, CA 95616, USA.
Email: tdkuo@ucdavis.edu
ORCID: 0000-0001-6167-0284

Supplemental Material

This PDF file includes:

Supplemental Figure S1-S7 (PFD)

Supplemental Table S1-S2 (PDF)

Other supplemental material for this manuscript includes the following:

Supplemental Dataset S1-S4 (XLS)

SUPPLEMENTAL FIGURES AND LEGENDS

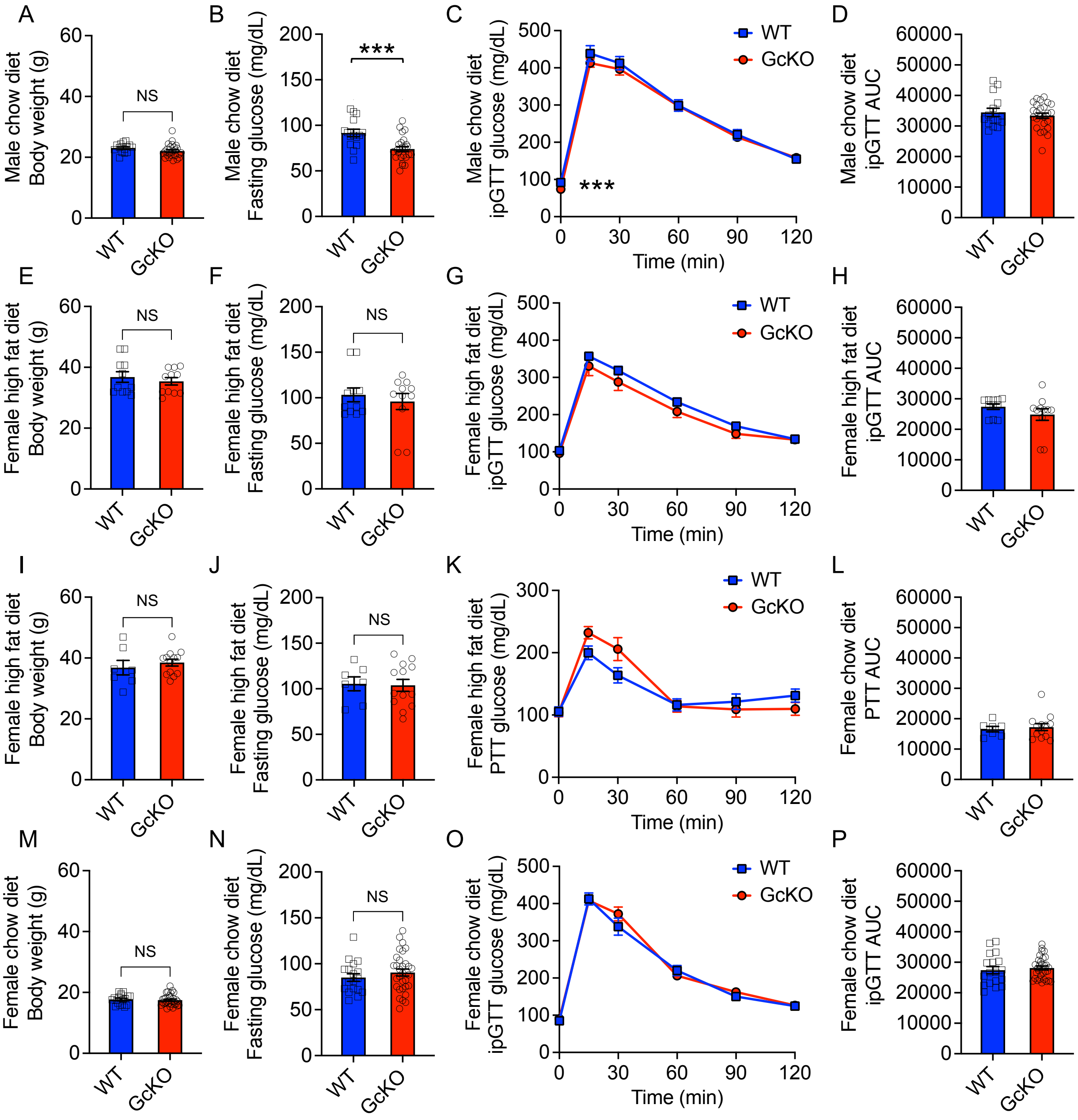

Figure S1. Metabolic characteristics of WT and GcKO male and female mice.

(A-D) Male mice on normal chow diet (NCD). **(A)** Body weight, **(B)** fasting glucose, **(C)** intraperitoneal glucose tolerance test (ipGTT), and **(D)** area under the curve (AUC) in C, in NCD-fed male WT (n= 15) and GcKO (n= 25) mice. **(E-H)** Female mice on high fat diet (HFD). **(E)** Body weight, **(F)** fasting glucose. **(G)** ipGTT, and **(H)** AUC in G, in HFD-fed female WT (n= 11) and GcKO (n= 11) mice. **(I-L)** Female mice on high fat diet (HFD). **(I)** Body weight, **(J)** Fasting glucose, **(K)** intraperitoneal pyruvate tolerance test (PTT), and **(L)** AUC in K, in HFD-fed female WT (n= 7) and GcKO (n= 13) mice. **(M-P)** Female mice on NCD. **(M)** Body weight, **(N)** fasting glucose, **(O)** ipGTT, and **(P)** AUC in K, in NCD-fed female WT (n= 18) and GcKO (n= 31) mice. "NS" indicates no significant statistical difference. Error bars represent ± SEM. Error bars represent ± SEM, *$p < 0.05$, **$p < 0.01$, ***$p < 0.005$ by Student's t test.

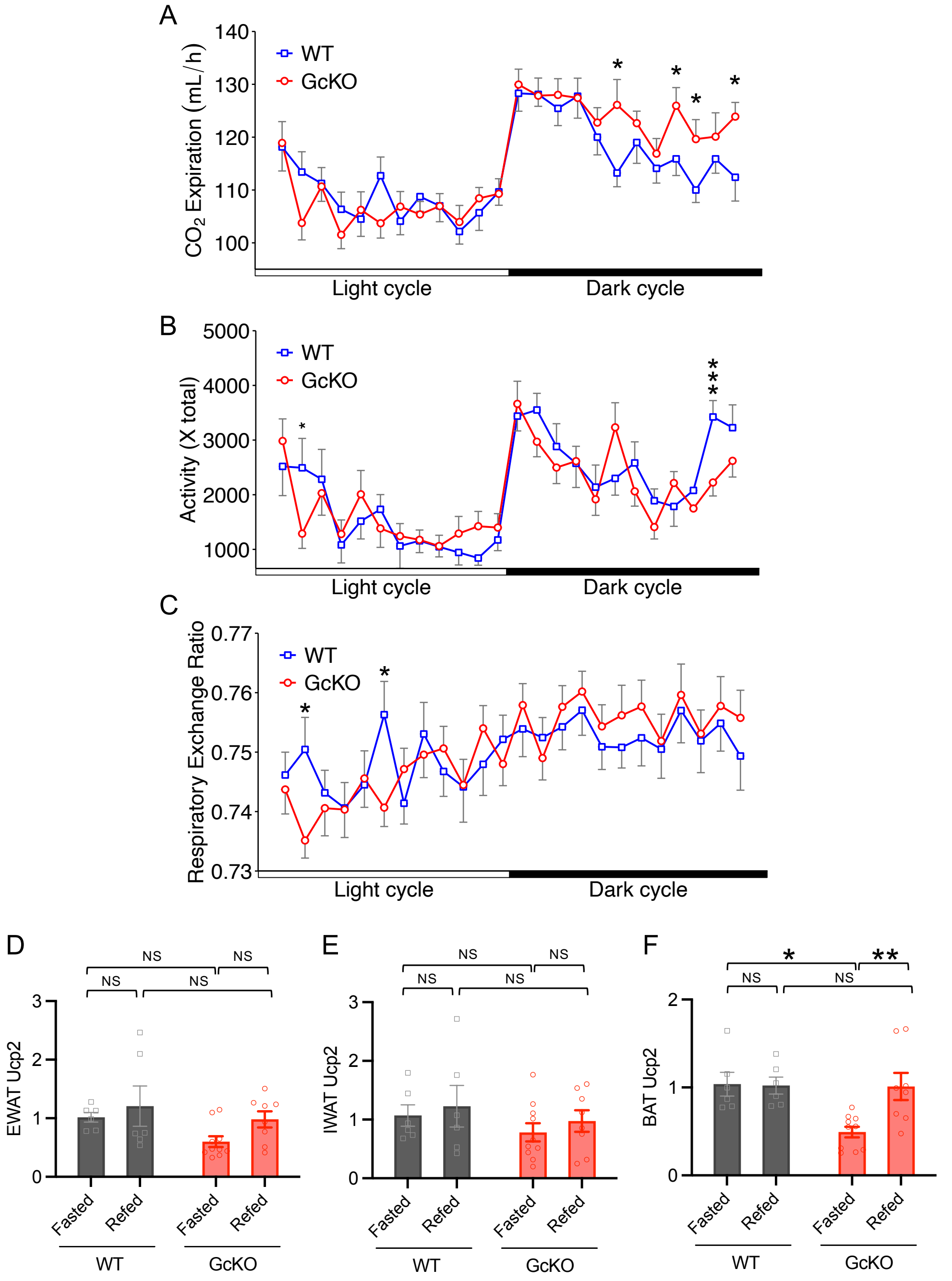

Figure S2. Indirect calorimetry shows increased energy expenditure in the absence of Gc.
A group of WT (n= 7) and GcKO (n= 7) male mice were subjected to indirect calorimetry analysis. Indirect calorimetry revealed carbon dioxide expiration per light and dark cycle **(A)**, activity per light and dark cycle **(B)**, and respiratory exchange ratio (RER) per light and dark cycle **(C)**. **(D-F)** Ucp2 gene expression in visceral/epidydimal white adipose tissues (EWAT) **(D)**, subcutaneous/inguinal white adipose tissues (IWAT) **(E)**, and brown adipose tissues (BAT) **(F)**. “NS” indicates no significance. Error bars represent ± SEM, *$p < 0.05$, **$p < 0.01$, ***$p < 0.005$ by Student’s t test in A, B, and C at different time points between genotypes, or two-way ANOVA in D, E, and F.

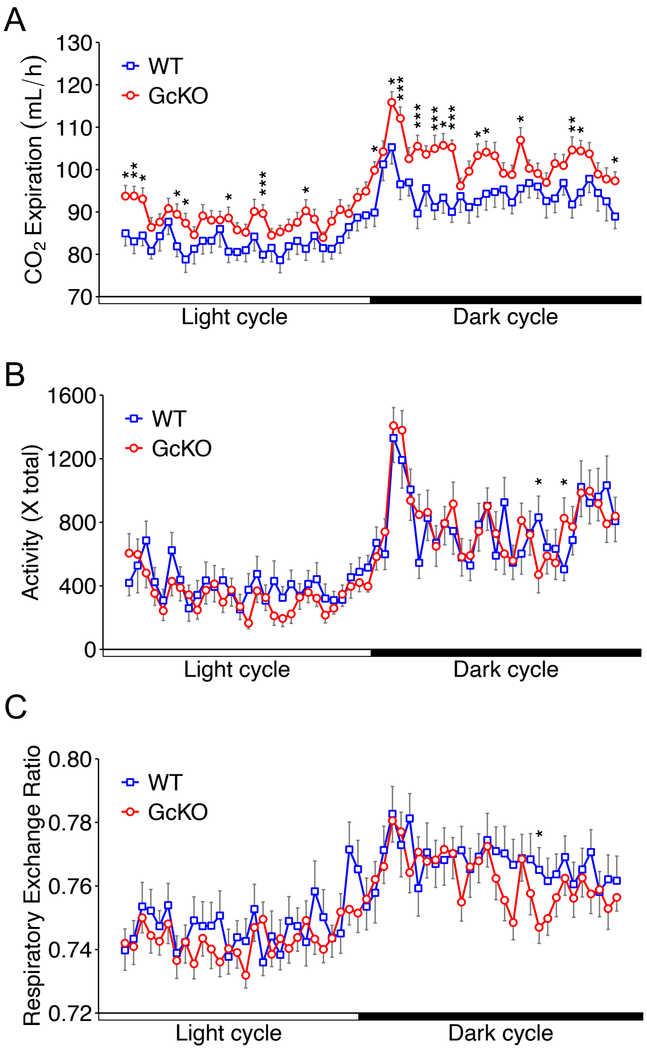

Figure S3. Indirect calorimetry shows increased energy expenditure in the absence of Gc, body weight-matched mice. A group of body weight-matched WT (n= 6) and GcKO (n= 9) male mice were subjected to indirect calorimetry analysis. Indirect calorimetry revealed carbon dioxide expiration per light and dark cycle **(A)**, activity per light and dark cycle **(B)**, and respiratory exchange ratio (RER) per light and dark cycle **(C)**. Error bars represent ± SEM, *$p < 0.05$, **$p < 0.01$, ***$p < 0.005$ by Student's t test. Student's t test was performed in A, B, and C at each time point between genotypes.

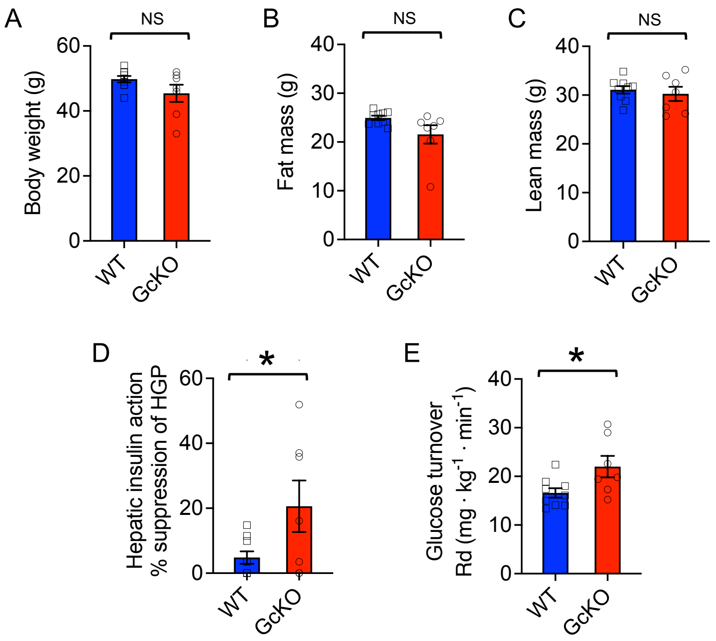

Figure S4. Hyperinsulinemic-euglycemic clamps in body weight- and fat mass-matched WT and GcKO mice. **(A)** Body weight, **(B)** fat mass, and **(C**) lean mass in WT and GcKO male mice subjected to hyperinsulinemic-euglycemic clamps. **(D)** Hepatic insulin action, calculated as the ratio of clamp hepatic glucose production (HGP) relative to basal HGP, and presented as percent suppression. **(E)** Glucose turnover rate, also known as rate of glucose disposal (Rd). WT n= 9, GcKO n= 7. "NS" indicates no significant statistical difference. Error bars represent $\pm$ SEM, *$p < 0.05$, **$p < 0.01$ by Student's *t* test.

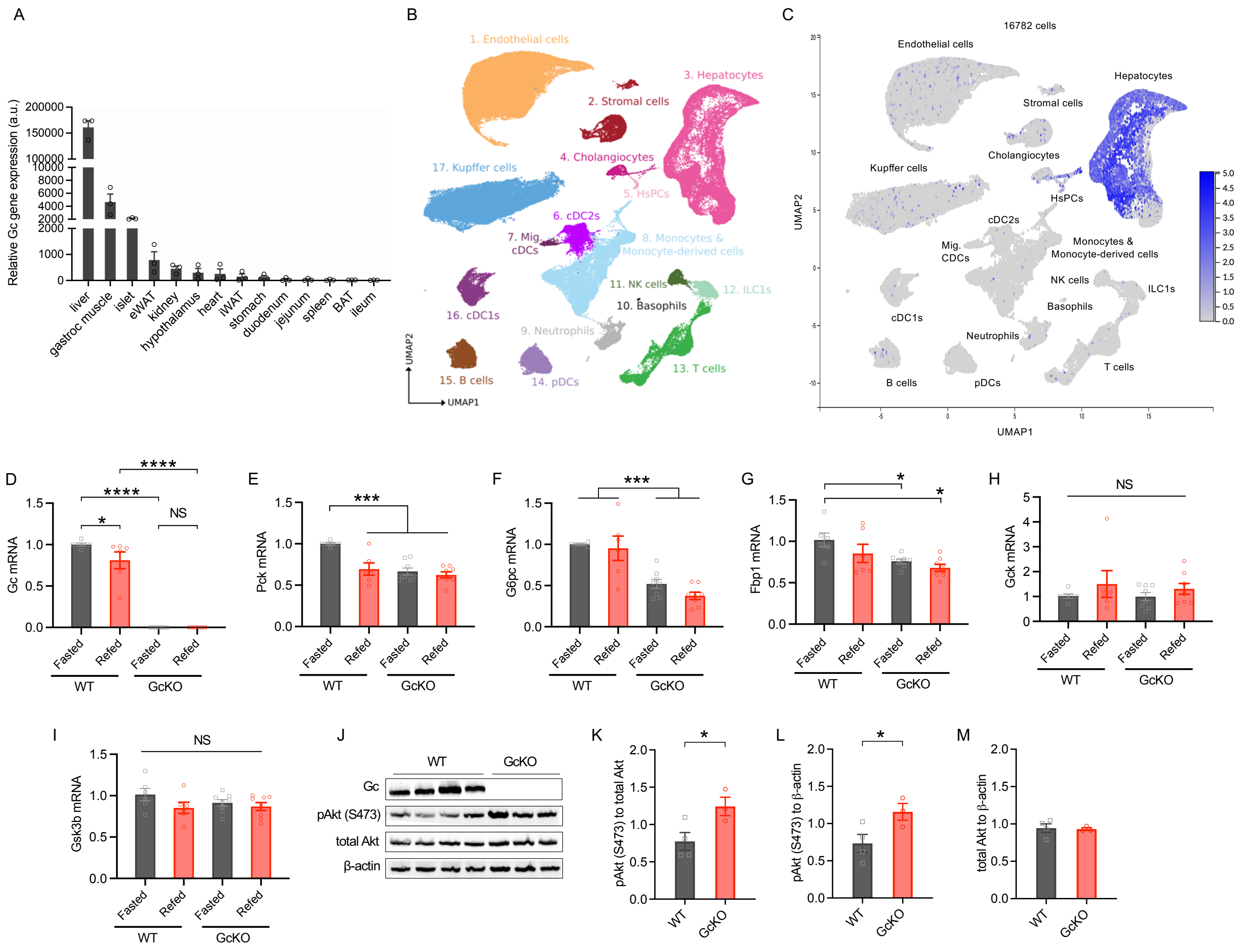

Figure S5. RNA and protein expression profiles in liver.

(A) Relative *Gc* expression in various metabolic tissues of WT mice.

(B) UMAP representing various cell types from single-cell CITE-seq in mouse liver. Data adapted from Liver Cell Atlas (Guilliams et al, 2022).

(C) Gc mRNA expression in various cell types in B. Data adapted from Liver Cell Atlas (Guilliams et al, 2022).

(D) *Gc* expression in fasted and refed WT and GcKO mice.

(E-G) Gluconeogenic genes *Pck1* (E), *G6pc* (F), and *Fbp1* (G) expression in fasted or refed WT and GcKO mice.

(H) Gck expression in fasted or refed WT and GcKO mice.

(I) Gsk3β expression in fasted or refed WT and GcKO mice. WT n= 6; GcKO n= 8.

(J) Immunoblots showing Gc and insulin signaling substrates in HFD-fed WT and GcKO mice.

(K) Quantification of normalized phosphorylated Akt at serine 473 to normalized total Akt in HFD-fed WT or GcKO mice in J.

(L) Quantification of phosphorylated Akt at serine 473 to beta-actin in HFD-fed WT or GcKO mice in J.

(M) Quantification of total Akt to beta-actin in HFD-fed WT or GcKO mice in J.

Mice are males. "NS" indicates no significant statistical difference. Error bars represent ± SEM, *$p < 0.05$, **$p < 0.01$, ***$p < 0.005$, ****$p < 0.001$ by Student's t test in K, L , and M, or two-way ANOVA in D, E, F, G, H, and I.

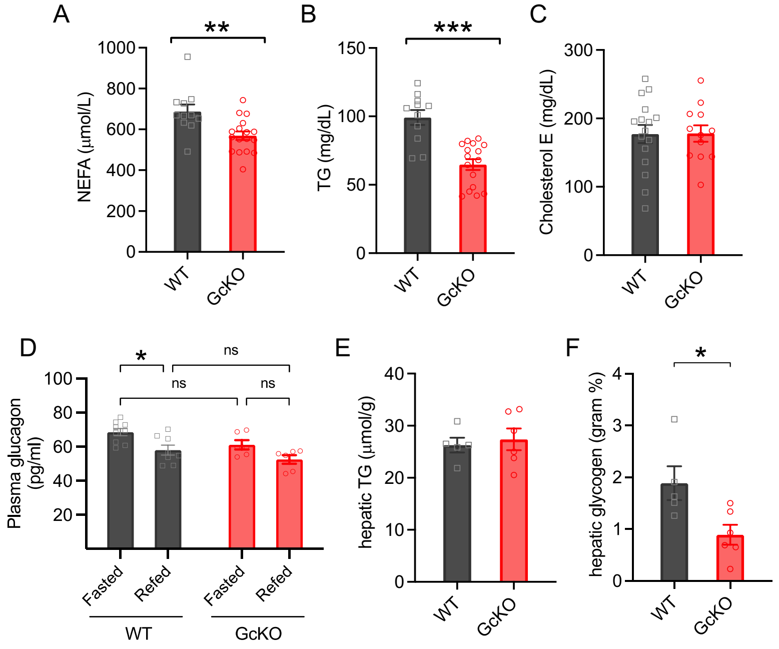

Figure S6. Lipid profiles in GcKO mice.

(A-C) Plasma non-esterified fatty acid (NEFA) (**A**), triglyceride (TG) **(B)**, and cholesterol E levels **(C)** in HFD-fed WT and GcKO mice. WT n $\geq$ 11, GcKO n $\geq$ 12. **(D)** Plasma glucagon levels in fasted and refed WT and GcKO mice. WT n= 8, GcKO n= 6. **(E)** Post-clamp hepatic TG content. **(F)** Post-clamp hepatic glycogen content. WT n= 5, GcKO n= 6. “NS” indicates no significant statistical difference. Error bars represent $\pm$ SEM, *$p < 0.05$, **$p < 0.01$, ***$p < 0.005$ by Student’s t test in A, B, C, E, and F, or two-way ANOVA in D.

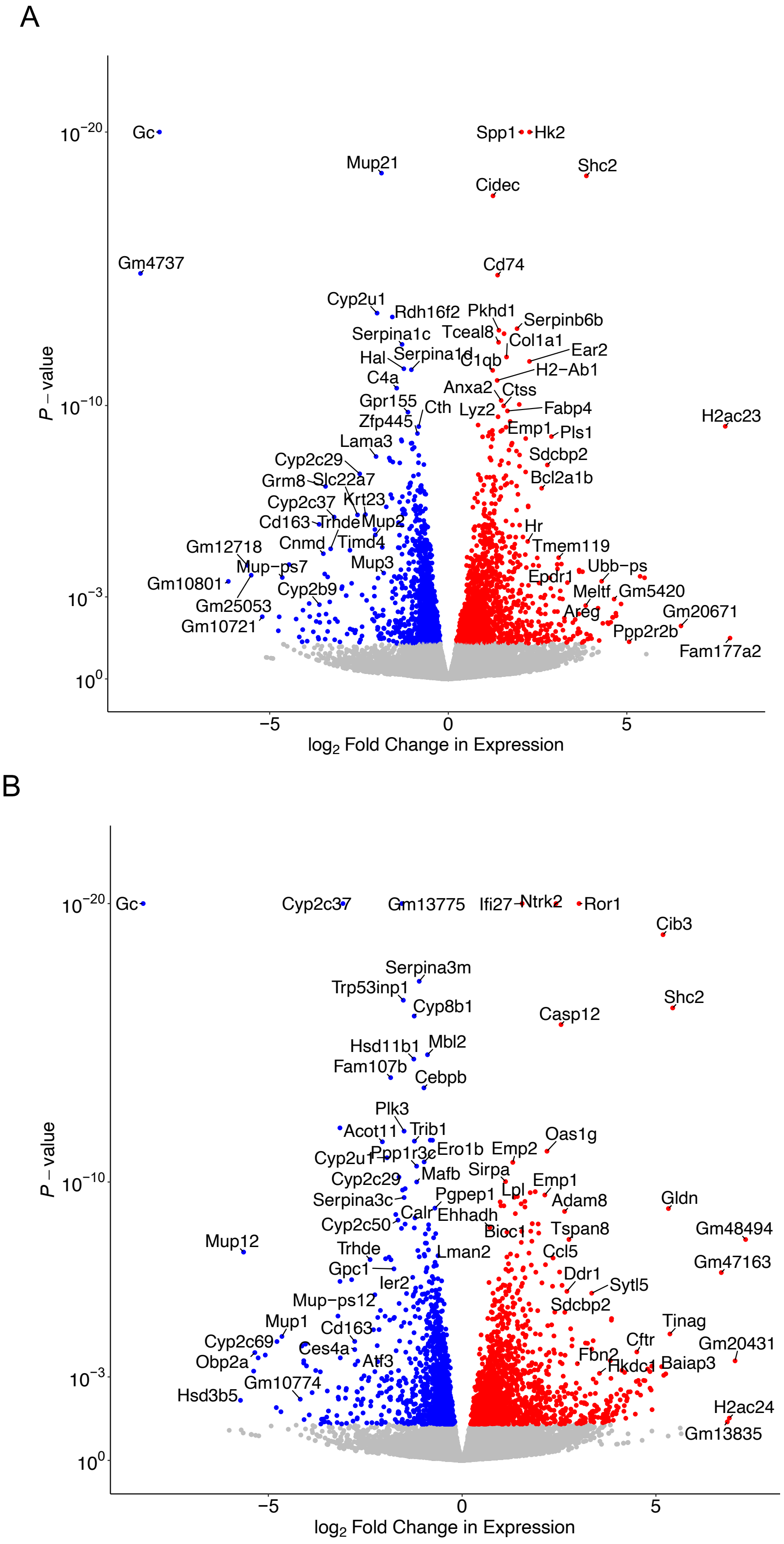

Figure S7. Genome-wide gene expression analysis in fasted and refed WT and GcKO liver.
WT and GcKO mice were either fasting for 16 h (fasted condition), or fasted for 16 h followed by 2 h refeeding (refed condition), and subjected for RNA-seq. Volcano plots show differentially expressed genes between **(A)** refed WT (n = 3) versus refed GcKO (n = 4), and **(B)** fasted WT (n= 3) versus fasted GcKO (n = 5) mice, where y-axes show negative $\log_{10}$ (*P* value), and x-axes represent $\log_2$ fold change in gene expression.

SUPPLEMENTAL TABLES

Supplemental Table S1: GC protein and its positive association with human diseases

Disease category	Disease	Individuals	Cases	P value
Diseases of the digestive system	Other or unspecified ileus, impaction or obstruction	32,275	593	0.0066917
Endocrine, nutritional and metabolic diseases	Disorders of lipoprotein metabolism and other lipidemias	40,372	6,075	0.0089938

Supplemental Table S2: Primer list

Mouse gene	primer sequence
Gc_F	GGCAGAGCTGAAAGACATGG
Gc_R	ATCAGGACTGCAGGGTGTCT
Pck_F	CCTGGAAGAACAAGGAGTGG
Pck_R	AGGGTCAATAATGGGGCACT
G6pc_F	GTCTGGATTCTACCTGCTAC
G6pc_R	AAAGACTTCTTGTGTGTCTGTC
Fbp1_F	TGCTGAAGTCGTCCTACGCTAC
Fbp1_R	TTCCGATGGACACAAGGCAGTC
Gck_F	TATGAAGACCGCCAATGTGA
Gck_R	TTTCCGCCAATGATCTTTTC
Gsk3b_F	GAGCCACTGATTACACGTCCAG
Gsk3b_R	CCAACTGATCCACACCACTGTC
Rpl19_F	TCCTTGGTCTTAGACCTGCG
Rpl19_R	ATGGAGCACATCCACAAGC
Ucp1_F	GCTTTGCCTCACTCAGGATTGG
Ucp1_R	CCAATGAACACTGCCACACCTC
Ucp2_F	CAGGTCACTGTGCCCTTACCA
Ucp2_R	AGGCATGAACCCCTTGTAGAAG
