# Supplementary material for: Gc inhibition preserves insulin sensitivity and reduces body weight without loss of muscle mass
Source: JCI Insight. 2025 Dec 8;10(23):e195341. doi: 10.1172/jci.insight.195341 (PMC12890482; doi:10.1172/jci.insight.195341)

Gill et al.,

Gc inhibition preserves insulin sensitivity and reduces body weight without loss of muscle mass

Figure 6C, unmodified gels from Bio-Rad ChemiDoc

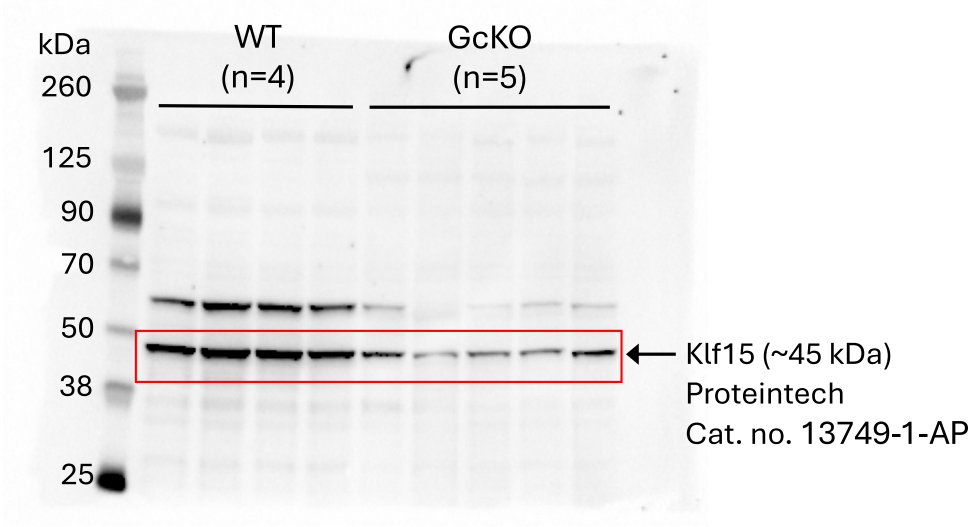

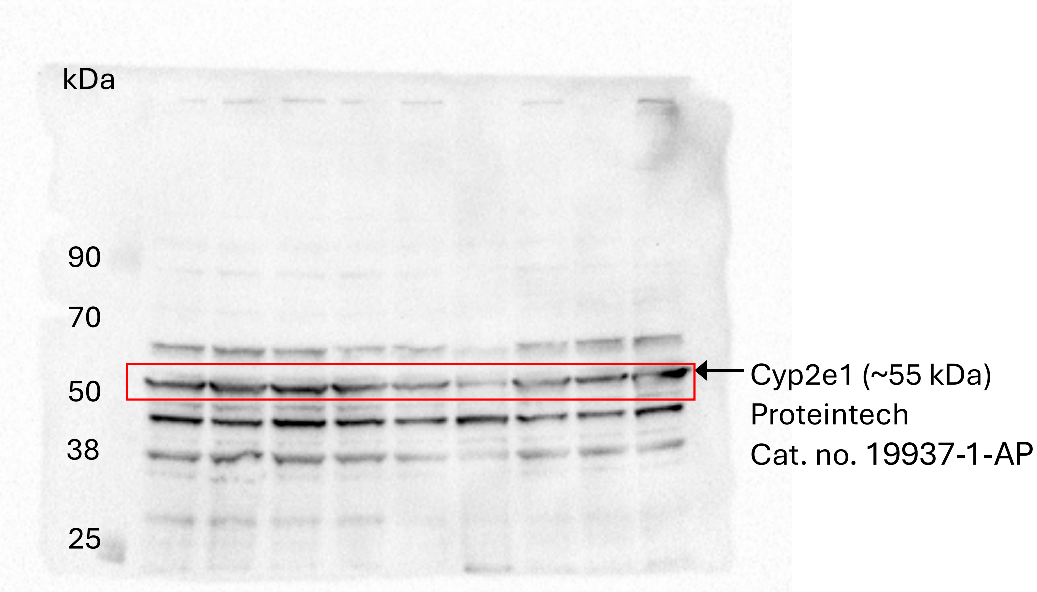

Gill et al.,
Gc inhibition preserves insulin sensitivity and reduces body weight without loss of muscle mass
Figure 6C, unmodified gels from Bio-Rad ChemiDoc

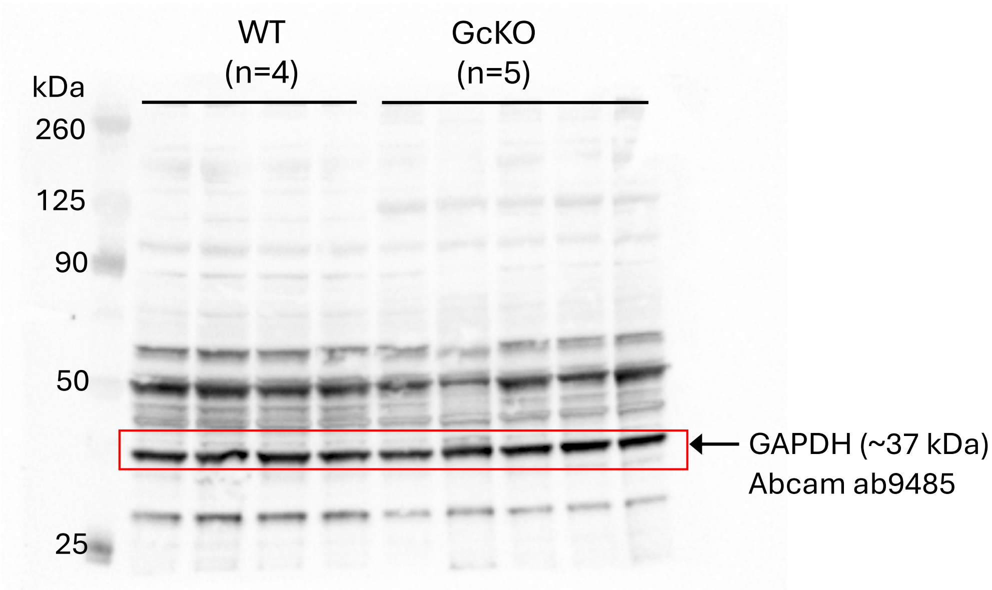

Gill et al.,
Gc inhibition preserves insulin sensitivity and reduces body weight without loss of muscle mass
Figure 7C, unmodified gels from LICOR

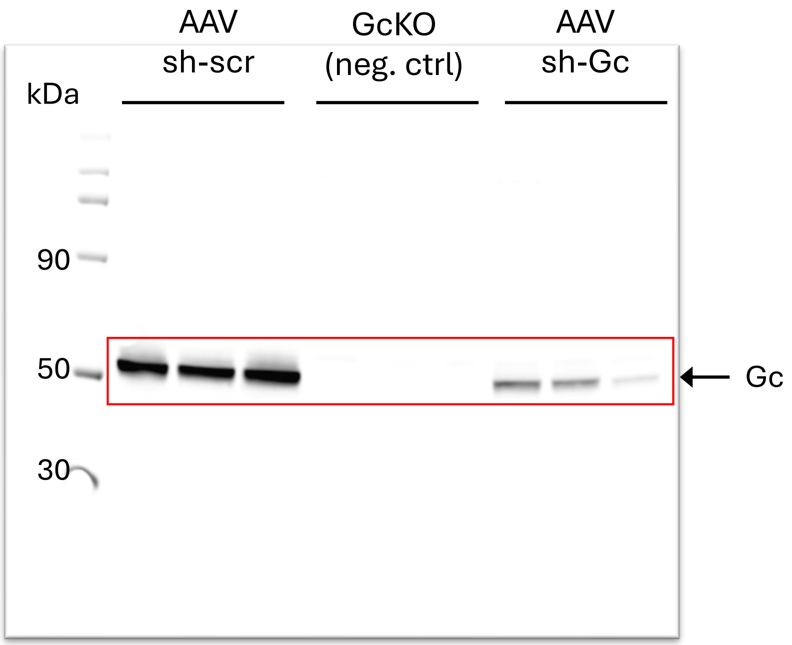

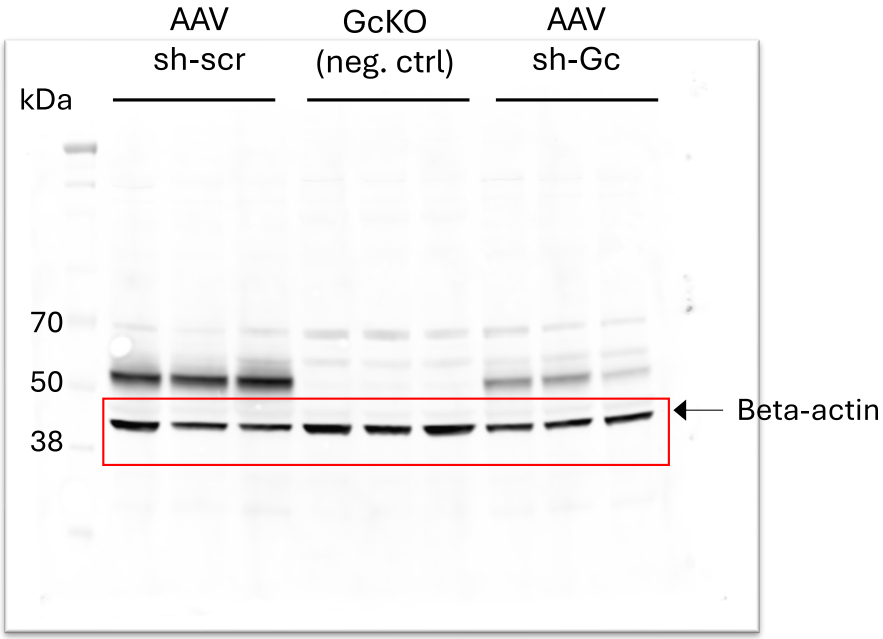

Gill et al.,
Gc inhibition preserves insulin sensitivity and reduces body weight without loss of muscle mass
Figure 7I, unmodified gels from LICOR

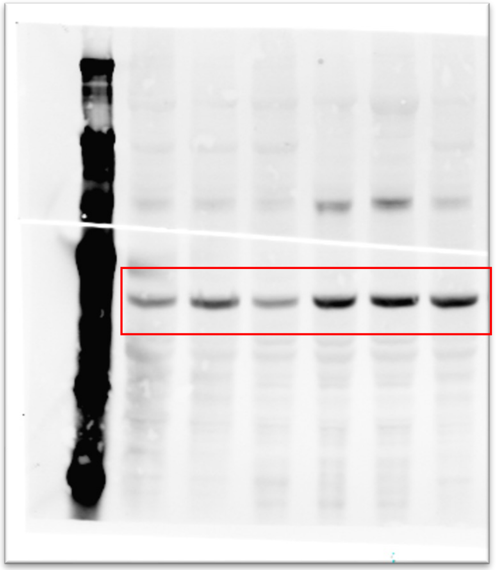

phospho-Akt
(S473)

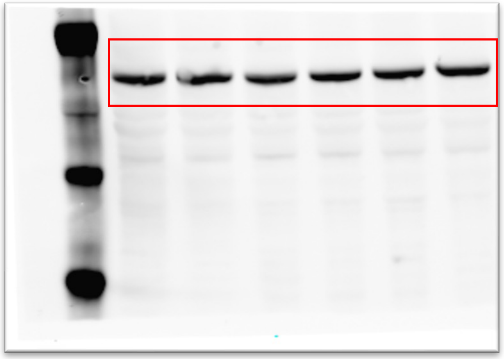

total Akt

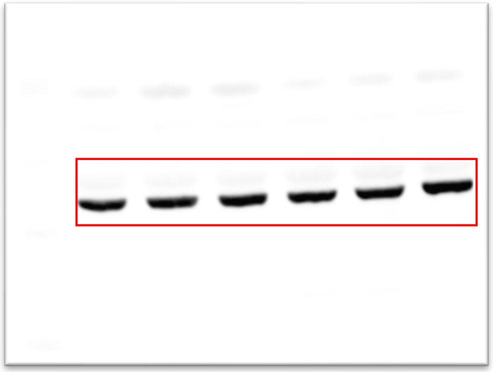

Beta-actin

Gill et al.,
Gc inhibition preserves insulin sensitivity and reduces body weight without loss of muscle mass
Figure S5J, unmodified gels from LICOR

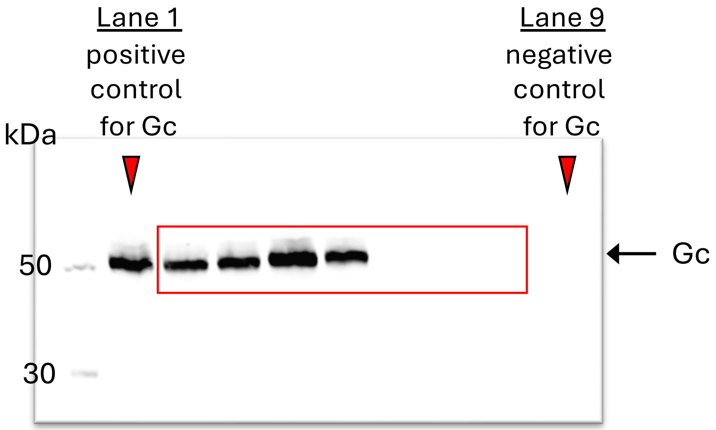

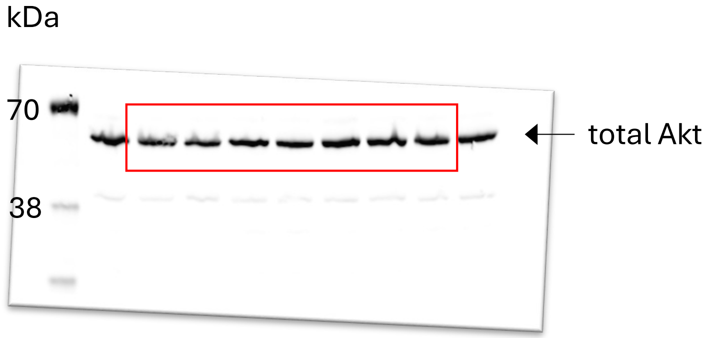

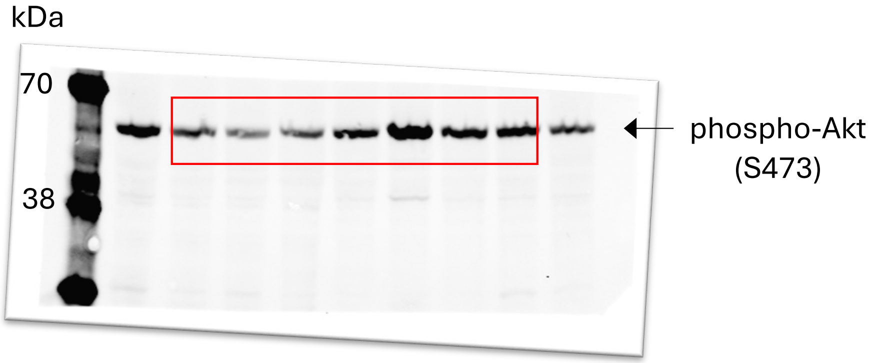

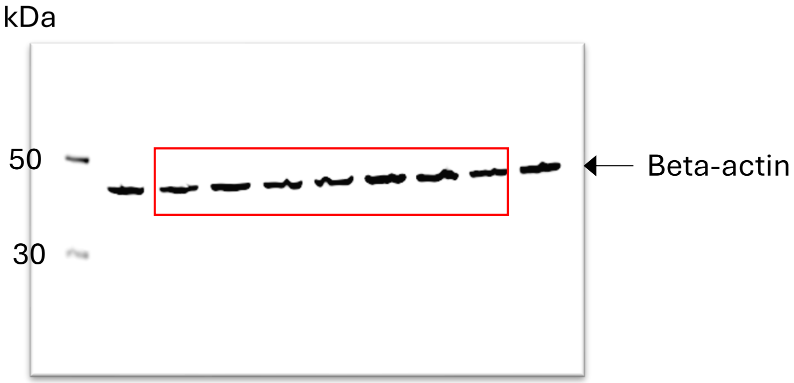

Supplement: Unedited blot and gel images [file jciinsight-10-195341-s251.pdf]
